# Supplementary material for: Systematic review of indoor residual spray efficacy and effectiveness against Plasmodium falciparum in Africa
Source: Nat Commun. 2018 Nov 26;9:4982. doi: 10.1038/s41467-018-07357-w (PMC6255894; doi:10.1038/s41467-018-07357-w)
Supplement: Supplementary file 1 — Supplementary Information [file 41467_2018_7357_MOESM1_ESM.pdf]

**Supplementary Information**

# Systematic review of indoor residual spray efficacy and effectiveness against *Plasmodium falciparum* in Africa

***Short title: Insecticidal spray efficacies for malaria and pyrethroid resistance***

Ellie Sherrard-Smith, Jamie T. Griffin, Peter Winskill, Vincent Corbel, Cédric Pennetier, A. Djénontin, Sarah Moore, Jason H. Richardson, Pie Müller, Constant Edi, Natacha Protopopoff, Richard Oxborough, Fiacre Agossa, Raphael N'Guessan, Mark Rowland, Thomas S. Churcher

## Supplementary Methods

A meta-analysis of IRS experimental hut trials is used to summarise measures of IRS efficacy. The meta-analysis was conducted based on the PRISMA guidelines which highlight how best to perform systematic reviews for clinical trial data. Here, we are interested in count data for mosquitoes in Phase II studies over a time series of multiple months. Four search engines were used (Web of Knowledge, PubMed, JSTOR and google scholar) to identify relevant data resources. Policy teams and author's regularly conducting these studies were also contacted to access unpublished resources. Supplementary Figure 1 schematically summarises the data search and extraction of key studies. Supplementary Table 1 notes those studies from which data are included in the subsequent analyses. The analyses are divided into 3 sections as outlined below.

### Analysis 1: Comparison of the initial impact of IRS products

Studies included are noted in Supplementary Table 1 and the data used for analysis 1 are listed in Supplementary Data 1. Analysis 1 was used to assess and compare the initial impact (within 2 months of spraying) of different IRS products. Supplementary Table 2 shows summary statistics for these general linear models of the predicted proportion of mosquitoes that are killed, exited, blood-fed or deterred by carbamates (bendiocarb), neonicotinoids (including clothianidin), organophosphates (including pirimiphos methyl) and pyrethroids (including alpha-cypermethrin, deltamethrin and lambda-cyhalothrin) after correcting for hut type (East or West African design), substrate (cement (including concrete) or mud) and mosquito species (*An. funestus, An. gambiae s.l.,* and *An. arabiensis*) (also see Figure 1 main manuscript).

**Analysis 2: Temporal characterisation of different active ingredients**

The data included in the temporal characterisation are noted in Supplementary Table 1. Reasons for excluding studies or resources are also reported here.

**Analysis 3: Pyrethroid resistance**

Studies included for the characterisation of pyrethroid resistance are recorded in Table 2 main manuscript. Reasons for excluding studies are noted in Supplementary Table 1.

**Supplementary Table 1:** The studies identified for full-text assessment for eligibility

| Study | Analysis 1: Comparison of the initial impact of IRS products (N = 28 included) | Analysis 2: Temporal characterisation of different active ingredients (N = 12 included) | Analysis 3: Pyrethroid resistance (bioassay and initial hut mortality, N = 13 included) | Analysis 3: Pyrethroid resistance (pyrethroid time series data) |
|---|---|---|---|---|
| 1. Akogbeto et al. 2010 [1] | Yes | Yes | Yes ([2] for bioassay mortality) | Yes (deltamethrin and alpha-cypermethrin) |
| 2. Agossa et al. 2014 [3] | Yes | Yes | Yes | Yes (lambda-cyhalothrin) |
| 3. Agossa et al. 2015 [4] | Yes | Yes | Yes | Yes (lambda-cyhalothrin, deltamethrin and alpha-cypermethrin) |
| 4. Agossa et al. 2018 [5] | Yes | Yes | Yes | Yes (deltamethrin) |
| 5. Asale et al. 2014 [6] | Yes | Four-week trial. Time series needed >= 2 months | No pyrethroid IRS experimental hut<br>Four-week trial. Time series needed >= 2 months | |
| 6. Barasa S., 2015 [7] | Yes | Single data point. Time series needed >= 2 months | No pyrethroid IRS experimental hut<br>Single data point. Time series needed >= 2 months | |
| 7. Chandre et al 2010 [8] | First time point over 2 months since spraying | Plastic sheeting used | | |
| 8. Diabate et al 2006 [9] | First time point over 2 months since spraying | Plastic sheeting used. | | |
| 9. Djènontin et al. 2010 [10] | Yes | Yes | No pyrethroid IRS experimental hut | |
| 10. Kitau et al 2014 [11] | Yes | 36 nights. Time series needed >= 2 months | Yes | 36 nights. Time series needed >= 2 months |
| 11. Malima et al. 2017 [12] | Not spraying | Durable wall lining used | | |
| 12. Mosqueira et al. 2010 [13] | Not IRS | Insecticide paint used. These two studies present | | |

| | | | | |
|---|---|---|---|---|
| 13. Mosquiera et al. 2013 [14] | | the same experimental hut data | | |
| 14. N'Guessan et al 2006 [15] | Not IRS | Repellent products used. | | |
| 15. N'Guessan et al 2009 [16] | Yes | Chlorfenapyr for IRS, not including prior to WHO recommendation. | | |
| 16. N'Guessan et al 2010 [17] | Yes | Data presented as summary only, Time series needed >= 2 months. | Same data as [18] | Data presented as summary only, Time series needed >= 2 months. |
| 17. N'Guessan et al 2007 [18] | Yes | Time series needed >= 2 months. | Yes ([19] for bioassay mortality) | Time series needed >= 2 months. |
| 18. Ngufor et al. 2016 [20] | Yes | Yes | Yes | Yes (alpha-cypermethrin) |
| 19. Ngufor et al. 2017 [21] | Yes | Chlorfenapyr for IRS, not including prior to WHO recommendation. | | |
| 20. Ngufor et al. 2017 [22] | Yes | Yes | Yes (using cement measurement for consistency) | Yes (3 datasets for deltamethrin) |
| 21. Ngufor et al. 2011 [23] | Yes | Chlorfenapyr for IRS, not including prior to WHO recommendation. | | |
| 22. Ngufor et al. 2014 [24] | Yes | Durable lining and net wall hangings used. | | |
| 23. Ngufor et al. 2014 [25] | Yes | Net wall hangings used. | | |
| 24. Ogoma et al. 2014 [26] | Yes | Coils and DDT on mats not IRS directly. | | |
| 25. Okumu et al 2013 [27]<br>26. Okumu, F., Moore, S. 2012. [28] | Data summarised across 7 months. | Summary data only.<br>Ifakara huts very different to alternative experimental huts. | | |
| 27. Okumu 2012 [29] | Introducing new Ifakara huts, not testing IRS.<br>Ifakara huts very different to alternative experimental huts. | | | |
| 28. Oxborough et al. 2010 [30] | Yes | No – *An. arabiensis*, too few data to include | Yes ([31] for bioassay mortality) | Yes |
| 29. Oxborough et al. 2014 [32] | Yes | Yes | No pyrethroid IRS experimental hut | |
| 30. Oxborough et al. 2014 [33] | Yes | No – *An. arabiensis*, too | Yes | Yes (2 surfaces) |

| | | | | |
|---|---|---|---|---|
| | | few data to include | | |
| 31. Randriamaherijaona et al. 2017 [34] | Yes | Fundamental differences in mosquito species in Madagascar relative to mainland Africa | | |
| 32. Randriamaherijaona et al. 2016 [35] | Yes | Fundamental differences in mosquito species in Madagascar relative to mainland Africa | | |
| 33. Rowland et al. 2013 [36] | Yes | Yes | Yes | Yes (alpha-cypermethrin) |
| 34. Tchicaya et al. 2014 [37] | Yes | Yes | Yes | Yes (lambda-cyhalothrin) |
| 35. World Health Organization 2002 Report of the 6th WHOPES working group. 2002. Geneva, Switzerland [38] | Summary review, no data (references extracted noted already) | | | |
| 36. World Health Organization 2013 Report of the 16th WHOPES working group meeting. 2013. Geneva, Switzerland [39] | Summary review, no data (references extracted noted already) | | | |
| 37. World Health Organization 2012 WHO Global Plan for insecticide resistance management in malaria vectors. 2012 Geneva, Switzerland [40] | Summary review, no data (references extracted noted already) | | | |
| 38. Yeebiyo et al. 2016 [41] | Only mortality data presented. | | | |
| 39. unpublished data Vincent Corbel | Yes | Yes | Yes | Yes (deltamethrin) |
| 40. unpublished data Pie Müller | Yes | Yes | No pyrethroid IRS experimental hut | |
| 41. unpublished data Sarah Moore | Yes | Ifakara huts very different to alternative experimental huts. | | |

Studies included or excluded from analysis 1-3 are noted with reasons for any exclusions.

**Supplementary Table 2:** Summary statistics for binomial logistic regression models

| | *Mortality(N = 78)* | | *Exophily (N = 74)* | | *Blood-feeding (N = 78)* | | *Deterrence (N = 78)* | |
|---|---|---|---|---|---|---|---|---|
| Covariate: *levels* | Coefficient (median; sd) | Uncertainty interval for posterior estimate | Coefficient (median; sd) | Uncertainty interval for posterior estimate | Coefficient (median; sd) | Uncertainty interval for posterior estimate | Coefficient (median; sd) | Uncertainty interval for posterior estimate |
| Intercept | -0.70 (0.11) | | 0.10 (0.18) | | 0.83 (0.12) | | -0.49 (0.12) | |
| Chemical: | | | | | | | | |
| *Carbamate* | (ref) | (ref) | (ref) | (ref) | (ref) | (ref) | (ref) | (ref) |
| *Neonicotinoid* | 0.76 (0.10) | 0.60 – 0.91 | -0.91 (0.10) | -1.07 – -0.74 | -0.18 (0.13) | -0.90 – -0.52 | -0.22 (0.13) | -0.32 – -0.50 |
| *Organophosphate* | 2.51 (0.09) | 2.36 – 2.66 | -0.49 (0.09) | -0.64 – -0.35 | -0.51 (0.12) | -0.71 – -0.33 | -0.04 (0.12) | -0.13 – 0.05 |
| *Pyrethroid* | 0.15 (0.09) | 0.01 – 0.30 | 0.77 (0.09) | 0.63 – 0.92 | -0.70 (0.12) | -0.41 – -0.03 | 0.41 (0.12) | 0.32 – 0.12 |
| Mosquito species: | | | | | | | | |
| *An. arabiensis* | (ref) | (ref) | (ref) | (ref) | (ref) | (ref) | (ref) | (ref) |
| *An. funestus* | -0.17 (0.16) | -0.42 – -0.09 | 2.29 (0.26) | 1.85 – 2.74 | 0.07 (0.13) | -0.14 – 0.28 | -1.20 (0.13) | -1.33 – -1.08 |
| *An. gambiae* | -1.24 (0.14) | -1.48 – -1.01 | 2.86 (0.26) | 2.43 – 3.31 | 0.39 (0.12) | 0.19 – 0.59 | -0.78 (0.12) | -0.90 – -0.67 |
| Hut design: | | | | | | | | |
| *East African* | (ref) | (ref) | (ref) | (ref) | (ref) | (ref) | (ref) | (ref) |
| *West African* | 1.80 (0.12) | 1.60 – 2.01 | -2.79 (0.20) | -3.14 – -2.48 | 0.52 (0.11) | 0.33 – 0.69 | 0.74 (0.11) | 0.64 – 0.84 |
| Wall substrate: | | | | | | | | |
| *Cement* | (ref) | (ref) | (ref) | (ref) | (ref) | (ref) | (ref) | (ref) |
| *Mud* | -0.10 (0.04) | -0.17 – -0.04 | -0.07 (0.03) | -0.12 – 0.02 | -0.25 (0.03) | -0.30 – -0.20 | 0.18 (0.03) | 0.15 – 0.21 |

Summary statistics for binomial logistic regression models fitted to the count data for mosquitoes that are killed, exited, blood-fed or deterred from experimental huts within 2 months since spraying (see Supplementary Figures 2-5 for visual confirmation of model fits). Here, the median coefficient for each model covariate is presented with the standard deviation and uncertainty intervals for the posterior estimates. These can be interpreted as the 0.9 probability of the coefficient for that covariate level to be between the presented range. A positive value is indicative of a higher proportion of mosquitoes being killed (mortality), exiting (exophily), blood-fed (blood-feeding) or probability of being in a treated compared to a control hut (indicative of deterrence) in the respective models relative to the baseline factor level defined.

**Model adjustments**

A widely used transmission dynamics model of malaria [42–45] is used to investigate the public health impact of different IRS compounds. In this model, people are born susceptible to *P. falciparum* infection and are exposed to infectious mosquito bites at a rate dependent on local mosquito density and infectivity. Maternal immunity is acquired for new born infants and this decays in the initial 6 months of life. Individuals are susceptible to clinical and severe disease and death after exposure [44,45]. The risk of developing infection declines with age due to naturally acquired immunity following continual exposure. Mosquito dynamics capture the effects of mosquito control and the resulting decline in egg laying [43]. A small number of minor changes (outlined below Supplementary Equations 1 – 22) are adopted to the IRS component of the model to reflect the varying impact of the new chemistries and how these change over time. These changes unify the way LLINs and IRS are represented in the model (and are parameterised with experimental hut trials) and provide greater flexibility to capture the impact of different insecticides.

**Change in insecticide efficacy over time**

Previous work has assumed that the efficacy of IRS decays at a constant rate and starts to decline immediately following spraying [42]. New longer-lasting products may have a more prolonged duration at maximum efficacy so new, more flexible decay curves are required to fully capture the impact of the insecticide. Previous mathematical models have also assumed that the endophily (the propensity for a mosquito to rest indoors following blood-feeding) remains constant when it may vary according to whether the house is sprayed with insecticides. New chemistries and the impacts of pyrethroid resistance mean that a spray may not kill a mosquito, but it may irritate it sufficiently so that it exits the hut without

blood-feeding. Here a flexible logistic function is used to capture the change in IRS impact over time since spraying ($t$, in days),

$$l_S = \frac{1}{1+\exp\left(-\left(l_{S\vartheta}+l_{S\gamma}\times t\right)\right)}, \quad \text{[S1]}$$

$$N_{dead} \sim binomial(l_s, N_{total1})$$

$$k_S = \frac{1}{1+\exp\left(-\left(k_{S\vartheta}+k_{S\gamma}\times t\right)\right)}, \quad \text{[S2]}$$

$$N_{successfully\_fed} \sim binomial(k_s, N_{total1})$$

$$m_S = \frac{1}{1+\exp\left(-\left(m_{S\vartheta}+l_{S\gamma}\times t\right)\right)}. \quad \text{[S3]}$$

$$N_{deterred} \sim binomial(m_s, N_{total2})$$

Matching previous notation (Griffin et al. 2010) the proportion of mosquitoes dying following entering a hut is denoted $l_S$ and is dependent on a parameter that determines initial efficacy ($l_{S\vartheta}$) and how this changes over time ($l_{S\gamma}$). The logistic model is fitted using the total number of mosquitoes killed ($N_{dead}$) in the sprayed huts ($N_{total1}$). Similarly, the proportion of mosquitoes successfully feeding ($k_S$) and being deterred away from a sprayed hut ($m_S$) is determined by the initial impact ($k_{S\vartheta}$ and $m_{S\vartheta}$, respectively) and how it changes with time ($k_{S\gamma}$ and $m_{S\gamma}$, respectively). Those mosquitoes that are deterred ($N_{deterred}$) are calculated as the number in the control huts minus those in the sprayed huts and the denominator here is the total mosquitoes in both control and treated huts ($N_{total2}$)._The percentage of mosquitoes that enter the hut and are repelled without being killed or feeding is then $j_s = 1 - l_s - k_s$. Previously the parameters for the transmission model have been fitted and estimate $k_0$ as 0.699. The $k_s$ fits are scaled to ensure that the probabilities that a mosquito entering a sprayed hut successfully blood-feeds, exits without feeding or dies, denoted $s_S$, $r_S$ and $d_S$ respectively, are within the appropriate 0 to 1 range

(Supplementary Figure 9). Supplementary Equations 1-3 were fitted simultaneously using Hamiltonian Monte Carlo sampling methods [46,47]. Four chains were initialised to assess the convergence of 2,000 iterations, the first 1,000 of each were discarded as burn in. The posterior distribution of parameters were then derived from the 4,000 iterations and 90% Bayesian credible intervals were estimated, posterior checks were performed using shinystan (version 1.0.0, [48]) and visually confirmed to fit the data. The parameter estimates for determining the impact of different IRS chemistries (pyrethroids, pirimiphos methyl, bendiocarb and clothianidin) are presented in Supplementary Table 3.

The probability that a mosquito is deterred away from a house with an intervention is typically estimated by the difference between total mosquito counts in experimental huts with and without interventions. This can be simply estimated for LLIN trials as the net can be rotated between huts therefore reducing any underlying hut heterogeneity in mosquito counts. The method is problematic for IRS trials because the walls of the hut are sprayed (and typically immovable) and so rotation is not possible. The pool of local mosquitoes nearest to sprayed huts is likely to both be spatially bias but also shrink over time due to the killing effect of IRS so that an artificial increase in the ratio of unsprayed to sprayed huts could be observed, spuriously indicating increased deterrence with time since spraying. It is biologically reasonable that the depreciation in any deterrence effect is broadly similar to the depreciation in the mortality effect (estimated by parameter $l_{S\gamma}$). Using this assumption, the initial deterrence is fitted using the data and then the same depreciation is assumed as for mortality (Supplementary Table 3).

The probabilities that a mosquito will be killed or successfully blood-fed are conditional on mosquitoes not being deterred before entering a sprayed hut. The functions $k_{s,}$ $l_s$ and $j_s$ are adjusted by the degree of deterrence ($m_s$, which is now a time varying quantity) as follows,

$$l'_S = l_S \times (1 - m_S) \quad \text{[S4]}$$

$$k'_S = k_S \times (1 - m_S) \quad \text{[S5]}$$

$$j'_S = j_S \times (1 - m_S) + m_S. \quad \text{[S6]}$$

Existing equations presented in Griffin et al (2010) for the per feeding attempt probability that a mosquito entering a sprayed hut successfully blood-feeds, exits without feeding or dies, denoted $s_S$, $r_S$ and $d_S$ respectively, are modified to account for this greater realism such that,

$$s_S = \frac{k'_S}{k_0} \quad \text{[S7]}$$

$$r_S = \left(1 - \frac{k'_S}{k_0}\right) \times \left(\frac{j'_S}{l'_S + j'_S}\right) \quad \text{[S8]}$$

$$d_S = \left(1 - \frac{k'_S}{k_0}\right) \times \left(\frac{l'_S}{l'_S + j'_S}\right). \quad \text{[S9]}$$

Following these changes, the model is consistent with Griffin et al. (2010). A full list of revised parameters with their definitions is provided in Supplementary Table 3.

**Supplementary Table 3:** Parameter estimates for the adjusted transmission dynamics model

| Parameter | Description | Estimate | | | |
|---|---|---|---|---|---|
| $k_0$ [ǂ] | Proportion of females bloodfed with no net | 0.699 | | | |
| $\Phi_I$ [ǂ] | Proportion of bites that are taken inside | An. funestus = 0.98<br>An. gambiae = 0.97<br>An. arabiensis = 0.96 | | | |
| $\Phi_B$ [ǂ] | Proportion of bites that are taken in bed | An. funestus = 0.9<br>An. gambiae = 0.89<br>An. arabiensis = 0.9 | | | |
| | | Pyrethroid[‡] | Actellic® (maximum and minimum range) | Bendiocarb (maximum and minimum range) | SumiShield® (maximum and minimum range) |
| $l_{S\vartheta}$ | Determines maximum killing effect for a given IRS | $\mu_{S\vartheta}$ = -1.060 (-1.14 – -0.98)<br>$\mu_{S\gamma}$ = 0.024 (0.02 – 0.03)<br>$\alpha_{l1}$ = -2.588<br>$\alpha_{l2}$ = 5.777 | 2.025<br>(1.832 – 4.754) | 1.095<br>(0.535 – 2.065) | 0.792<br>(-0.127 – 2.035) |
| $l_{S\gamma}$ | Determines how quickly IRS killing effect wanes | $\alpha_{l3}$ = -0.003<br>$\alpha_{l4}$ = -0.014 | -0.009<br>(-0.014 – -0.010) | -0.025<br>(-0.026 – -0.030) | -0.007<br>(-0.006 – -0.010) |
| $k_{S\vartheta}$ | Determines maximum blood-feeding inhibition effect for a given IRS | $\beta_{S\vartheta}$ = 0.767 (0.69 – 0.85)<br>$\beta_{S\gamma}$ = -0.032 (-0.03 – -0.03)<br>$\alpha_{k1}$ = -2.955<br>$\alpha_{k2}$ = 5.231 | -2.222<br>(-2.167 – -4.360) | -1.278<br>(-0.464 – -2.051) | -1.382<br>(-0.556 – -2.481) |
| $k_{S\gamma}$ | Determines how quickly IRS blood-feeding inhibition | $\alpha_{k3}$ = 0.012<br>$\alpha_{k4}$ = -0.005 | 0.008<br>(0.014 – 0.006) | 0.020<br>(0.016 – 0.023) | 0.009<br>(0.007 – 0.011) |

| | | | | | |
|---|---|---|---|---|---|
| | effect wanes | | | | |
| $m_{S\vartheta}$ | Determines maximum deterrence effect for a given IRS | $\varepsilon_{S\vartheta}$ = -1.674 (-1.79 – -1.57)<br>$\varepsilon_{S\gamma}$ = -0.001 (-0.003 – 0.001)<br>$\alpha_{m1}$ = -3.918<br>$\alpha_{m2}$ = 8.543 | -1.232<br>(-4.471 – -1.000) | -1.697<br>(-1.531 – -1.3413) | -0.458<br>(-0.092 – -1.445) |
| $m_{S\gamma}$ | Determines how quickly IRS deterrence effect wanes (fixed to $l_{S\gamma}$) | $\alpha_{m3}$ = 0.003<br>$\alpha_{m4}$ = -0.005 | -0.009 | -0.025 | -0.007 |

ƚ Taken from Griffin et al. (2010) all other parameters were estimated in the analysis for this manuscript.

‡ Pyrethroid parameters are dependent on the level of pyrethroid resistance (see Supplementary Equations 10-22).

Values indicate the best fit mean parameter values for each type of insecticide applied using indoor residual spraying (IRS). Parameters for pyrethroid IRS are dependent on the level of pyrethroid resistance in the mosquito population as measured by mortality in a discriminatory dose bioassay (see Supplementary Equation 10 in the main text). No insecticide resistance was assumed for other IRS products (Actellic®, Bendiocarb or SumiShield®).

There is a lot of variation in the measured performance of chemistries across experimental hut studies (Figure 1 main manuscript and Supplementary Figure 6). To capture this, the above functions are fitted to the best and worst performing data for each chemistry independently to estimate a range in product performance. These upper and lower bounds are provided in Table S3. The maximum and minimum cases averted per person per year that are estimated by the best and worst performing experimental hut time series, for each administration unit across Africa, are presented alongside the predictions determined by using the average parameter set to provide the expected range in predicted impact of IRS (Supplementary Data 2).

To test the utility of these IRS fits, the parameters are used to recreate RCTs on Actellic®300CS and bendiocarb (Figure 4 main manuscript). Each individual study parameter set is used to predict the performance of the IRS product, the study specific parameters are provided in Supplementary Table 4.

**Supplementary Table 4:** Individual study parameterisations for the randomised control trials re-created using the experimental hut data

| | Parameter estimates | | | | | |
|---|---|---|---|---|---|---|
| Study, number refers to Table 2 main text | $l_{S\vartheta}$ | $l_{S\gamma}$ | $k_{S\vartheta}$ | $k_{S\gamma}$ | $m_{S\vartheta}$ | $m_{S\gamma}$ |
| Actellic | | | | | | |
| 1[3] | 4.578 | -0.025 | -4.877 | 0.026 | -1.029 | -0.025 |
| 5[37] | 2.038 | -0.009 | -2.489 | 0.009 | -1.357 | -0.009 |
| 4[36] | 5.118 | -0.014 | -5.240 | 0.014 | -1.974 | -0.014 |
| 12* | 2.328 | -0.016 | -2.637 | 0.016 | 0.015 | -0.016 |
| 13[36ƚ] | 1.893 | -0.007 | -1.913 | 0.006 | -1.787 | -0.007 |
| 9[32] | 1.843 | -0.003 | -2.323 | 0.001 | 11.314 | -0.003 |
| Bendiocarb | | | | | | |
| 1[3] | 4.151 | -0.088 | -1.678 | 0.028 | -1.469 | -0.088 |
| 6[49] | 2.382 | -0.017 | -3.496 | 0.003 | -2.074 | -0.017 |
| 11[‡] | 0.639 | -0.027 | -0.679 | 0.021 | -0.588 | -0.027 |
| 8[10] | 3.474 | -0.140 | -2.767 | 0.094 | -3.760 | -0.140 |

*Unpublished data 2 in Table 2 main text; [ƚ]Parameter estimates for mud walls rather than 4 which refers to cement walls; [‡]Unpublished data 1 in Table 2 main text

Notation matches that for Supplementary Table 3. Study numbers relate to studies in Table 2 main text.

**Pyrethroid resistance**

Discriminating dose bioassays (WHO tube assay, WHO cone assay, CDC bottle assay) are a practical option for control programmes to assess the level of insecticide resistance. Although the simple bioassay has its limitations [50,51] it provides a useful measure to link the severity of mosquito insecticide resistance estimated in the field to the results of experimental hut trials evaluating new products [50,52]. The concentration of insecticide used in the discriminatory dose bioassay varies with the type of pyrethroid insecticides used. There were 18 data points identified in the meta-analysis where pyrethroid bioassays were

conducted on the same mosquito population as the experimental hut studies using a pyrethroid IRS (Supplementary Data 1 – analysis 3a). There were a further 21 datasets with time series data, but not bioassay mortality data (Supplementary Data 1 – analysis 3b), so that the initial (time *t = 0 days*) mosquito mortality, successful feeding and exiting probabilities and how the impact of pyrethroid IRS on mosquito behaviour changes over time could be estimated. This is insufficient data to differentiate between different types of pyrethroid, different mosquito species or wall substrates, so all pyrethroid data are pooled together. Let $x$ be the proportion of mosquitoes dying in a standard pyrethroid discriminatory dose bioassay. A test for the level of pyrethroid resistance in the mosquito population (expressed as a percentage, denoted $I$) is described by the following equation,

$$I = 100 \times (1 - x). \quad [S10]$$

The impact of pyrethroid resistance on the parameters influencing IRS efficacy is estimated from the 18 studies where concurrent bioassay and experimental hut data were collected (Figure 2a main manuscript). At the start of each trial, the mosquito mortality ($l_{S\,(t=0)}$), blood-feeding ($k_{S\,(t=0)}$) and deterrence ($m_{S\,(t=0)}$) measured in experimental huts can be associated with bioassay survival (as a measure of resistance) using,

$$l_{S(t=0)} = \frac{1}{1+\exp\left(-(\mu_{S\vartheta}+\mu_{S\gamma}\times x)\right)}, \quad [S11]$$

$$N_{dead} \sim binomial\left(l_{s(t=0)}, N_{total1}\right)$$

$$k_{S(t=0)} = \frac{1}{1+\exp\left(-(\beta_{S\vartheta}+\beta_{S\gamma}\times x)\right)}, \quad [S12]$$

$$N_{successfully\_fed} \sim binomial\left(k_{s(t=0)}, N_{total1}\right)$$

$$m_{S(t=0)} = \frac{1}{1+\exp\left(-(\varepsilon_{S\vartheta}+\varepsilon_{S\gamma}\times x)\right)}. \quad [S13]$$

$$N_{deterred} \sim binomial\left(m_{s(t=0)}, N_{total1}\right)$$

The change in mosquito mortality ($l_S$), blood-feeding ($k_S$) and deterrence ($m_S$) observed over time (Supplementary Equations 1-3) are estimated for each study separately. This generates 21 parameter sets describing how each trait changes over time. The relationship between these parameters can then be associated with mortality, blood-feeding and deterrence measured at time *t = 0* days such that,

$$l_{S\vartheta} = \alpha_{l1} + \alpha_{l2} l_{S\vartheta(t=0)}, \quad [S14]$$

$$l_{S\gamma} = \alpha_{l3} + \alpha_{l4} l_{S\vartheta(t=0)}, \quad [S15]$$

$$k_{S\vartheta} = \alpha_{k1} + \alpha_{k2} k_{S\vartheta(t=0)}, \quad [S16]$$

$$k_{S\gamma} = \alpha_{k3} + \alpha_{k4} k_{S\vartheta(t=0)}, \quad [S17]$$

$$m_{S\vartheta} = \alpha_{m1} + \alpha_{m2} m_{S\vartheta(t=0)}, \quad [S18]$$

$$m_{S\gamma} = \alpha_{m3} + \alpha_{m4} m_{S\vartheta(t=0)}, \quad [S19]$$

where $\alpha_{l1}$ to $\alpha_{l4}$ determine the shape of the relationships for mosquito mortality. Similarly, for $k_{S\vartheta}, k_{S\gamma}, \alpha_{k1-4}$ or $m_{S\vartheta}, m_{S\gamma}, \alpha_{m1-4}$ enables the impact of pyrethroid resistance on successful blood-feeding or deterrence to be quantified. Substituting in Supplementary Equation 11 to Supplementary Equations 14-15 (and similarly for equations Supplementary Equation 12 for 16-17 and Supplementary Equation 13 for 18-19) enables the relationship between prevalence, pyrethroid resistance and the change in IRS efficacy over time to be characterised and included within the mathematical model. These changes are demonstrated in Supplementary Figure 9. Following [42], the final probabilities determining mosquito behaviour in an area with LLINs and IRS are given in Supplementary Table 5 taking

into account the proportion of mosquitoes bites taken when people are indoors ($\Phi_I$) or in bed ($\Phi_B$). Further details of the statistical models fitted are shown below.

**Adjustments to bed net estimates for resistance**

Bed net estimations have been fitted to historic net use data using a transmission dynamic model previously [42,53]. Separately, the impact of insecticide resistance on bed nets has been previously quantified [50]. To ensure that the estimates in the current work adhered to these fitted calculations, a simple adjustment to the parameters determining the impact of pyrethroid resistance on nets was made as follows:

$$d_{n0,x} = d_{n0,x}/d_{n0,x=1} \times d_{n0,griffin} \quad \text{[S20]}$$

$$s_{n0,x} = s_{n,griffin} + (s_{n0,x} - s_{n0,x=0})/(s_{n0,x=1} - s_{n0,x=0}) \times (s_{n0,x=1} - s_{n0,griffin}) \quad \text{[S21]}$$

$$r_{n0,x} = 1 - d_{n0,x} - s_{n0,x} \quad \text{[S22]}$$

Where $d_{n0,x}$, $s_{n0,x}$ and $r_{n0,x}$ represent the probability that a mosquito is killed, fed successfully or repelled per feeding attempt at time $t = 0$ in the presence of insecticide resistance (x is the proportion of mosquitoes surviving a discriminatory dose bioassay test). The original parameters from Griffin et al. (2010) are $d_{n0,griffin}$ = 0.51 and $s_{n0,griffin}$ = 0.31.

All functions were fitted using Hamiltonian Monte Carlo sampling methods [46,47]. Four chains were initialised to assess the convergence of 2,000 iterations, the first 1,000 of each were discarded as burn in. The posterior distribution of parameters were then derived from the 4,000 iterations and 90% Bayesian credible intervals were estimated, posterior checks were performed using shinystan (version 1.0.0, [48]) and visually confirmed to fit the data. The

parameter estimates for determining the impact of different IRS chemistries (pyrethroids, pirimiphos methyl, bendiocarb and clothianidin) are presented in Supplementary Table 3.

**Supplementary Table 5:** Vector control probability matrix

| | IRS only | LLINs only | IRS plus LLIN |
|---|---|---|---|
| Probability of successful feeding | $1-\varphi_I+\varphi_I(1-r_S)s_S$ | $1-\varphi_B+\varphi_B s_N$ | $1-\varphi_I + \varphi_B(1-r_S)s_N s_S + (\varphi_I-\varphi_B)(1-r_S)s_S$ |
| Probability of biting | $1-\varphi_I+\varphi_I(1-r_S)$ | $1-\varphi_B+\varphi_B s_N$ | $1-\varphi_I + \varphi_B(1-r_S)s_N + (\varphi_I - \varphi_B)(1-r_S)$ |
| Probability of repellency | $\varphi_I r_S$ | $\varphi_B r_N$ | $\varphi_B(1-r_S)r_N+\varphi_I r_S$ |

Subscript *N* denotes how an LLIN net modifies the probability of successful blood-feeding or being repelled (see Griffin et al. 2010).

**Supplementary Figure 1:** The systematic review process

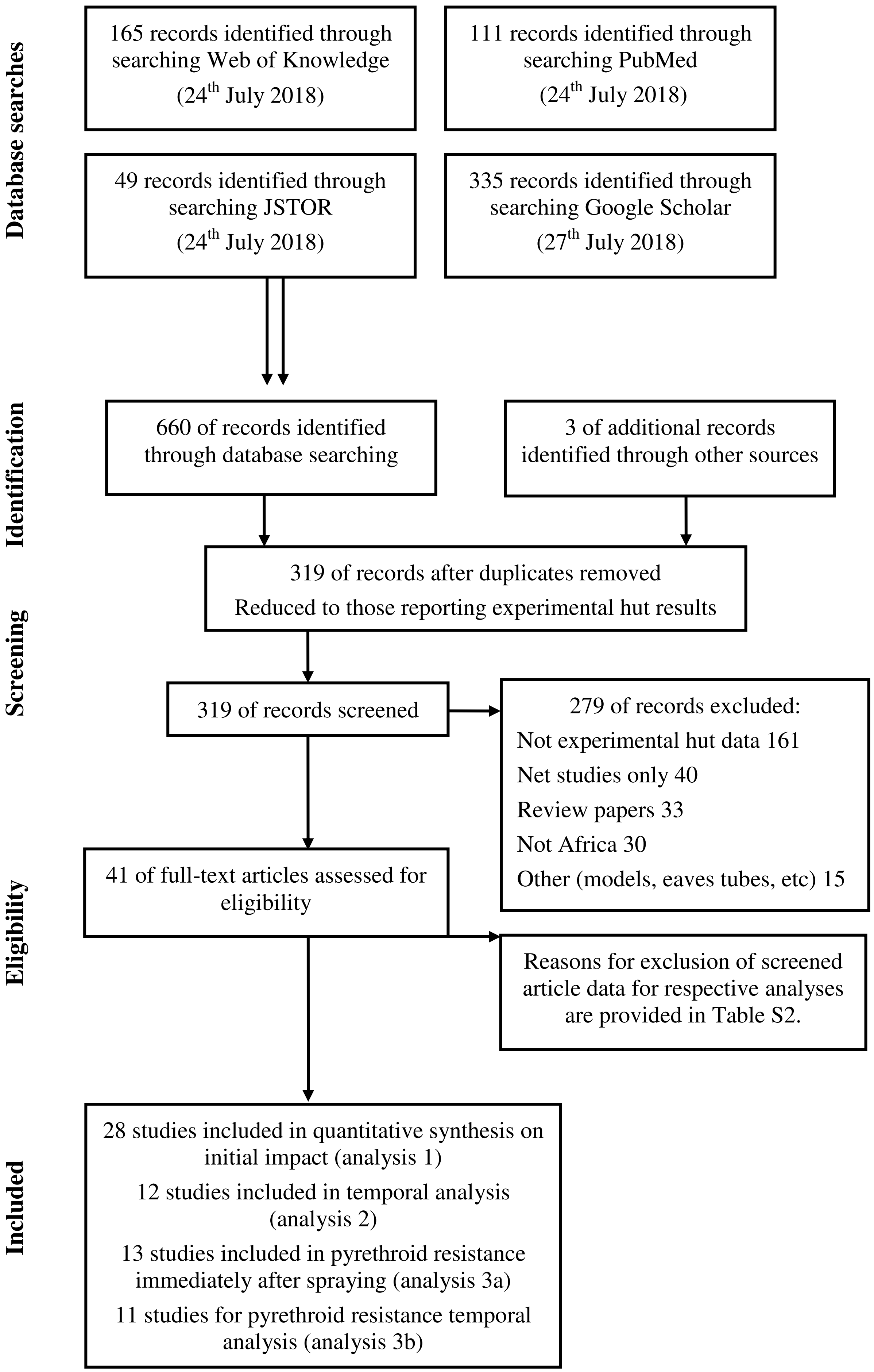

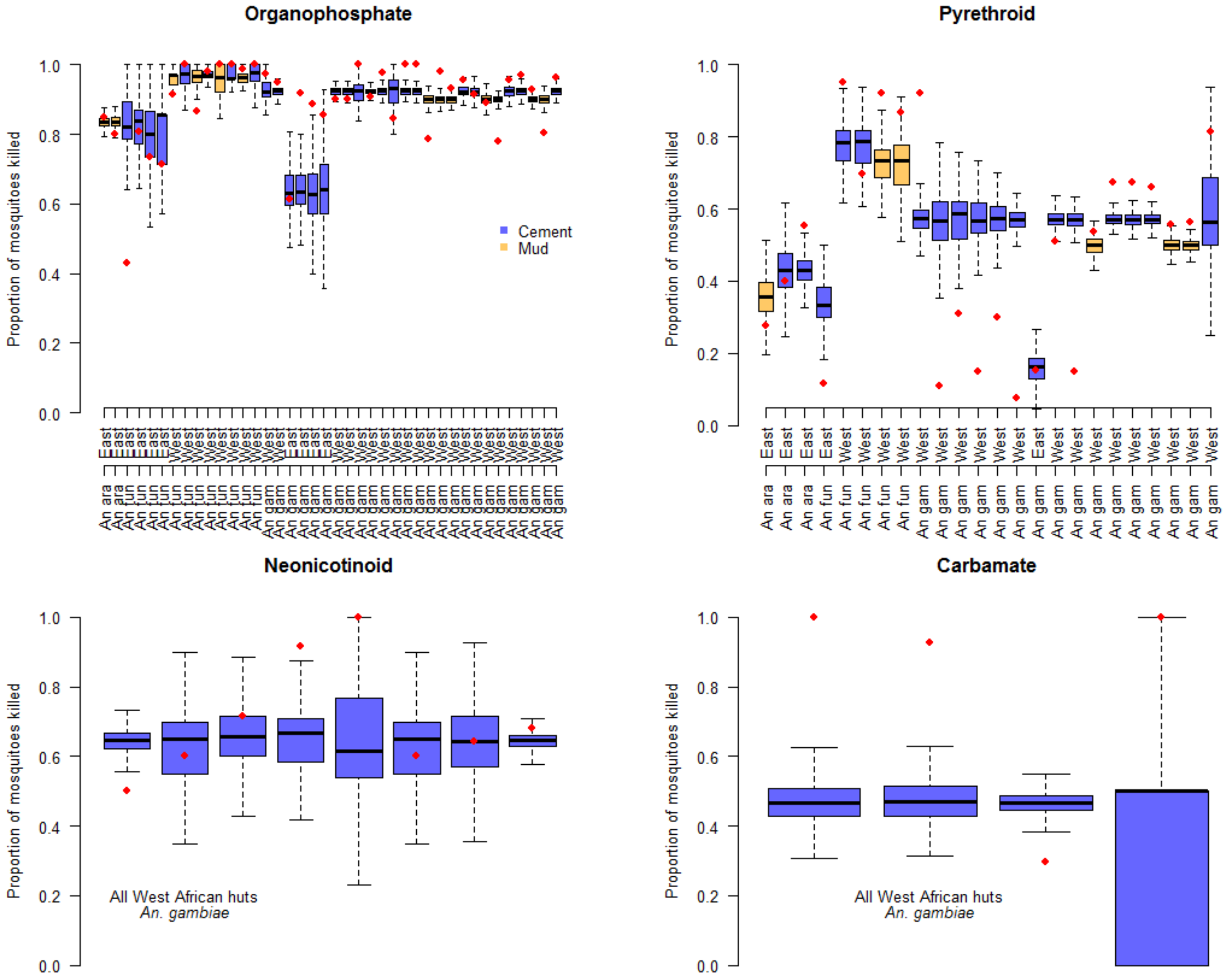


**Supplementary Figure 2: Bayesian model predictions for the proportion of mosquitoes that are killed in the sprayed experimental huts.** Chemistry classes are shown in each panel, model predictions are determined by mosquito species (An ara = *An. arabiensis*; An fun = *An. funestus*; An gam = *An. gambiae*), hut type (East or West African design[54]) and whether the substrate of the hut was made of mud, yellow, or cement (concrete walled-huts are included as cement), in blue. Boxplots show the median (line), 25$^{th}$ and 75$^{th}$ credible intervals, CrIs, (box) and 5$^{th}$ and 95$^{th}$ CrIs for the posterior predictive estimates of the proportion of mosquitoes that are killed by each chemistry. Red points indicate the actual data estimate. The proportion of mosquitoes that are killed by spraying in experimental huts is predicted to be greater in the presence of organophosphate relative to alternative chemistries, greater in West African huts than East African huts, greater on cement relative to mud walls, but relatively similar for different mosquito species. Note that there are minimal data for neonicotinoids and carbamates, *An. arabiensis* and mud-walled huts.

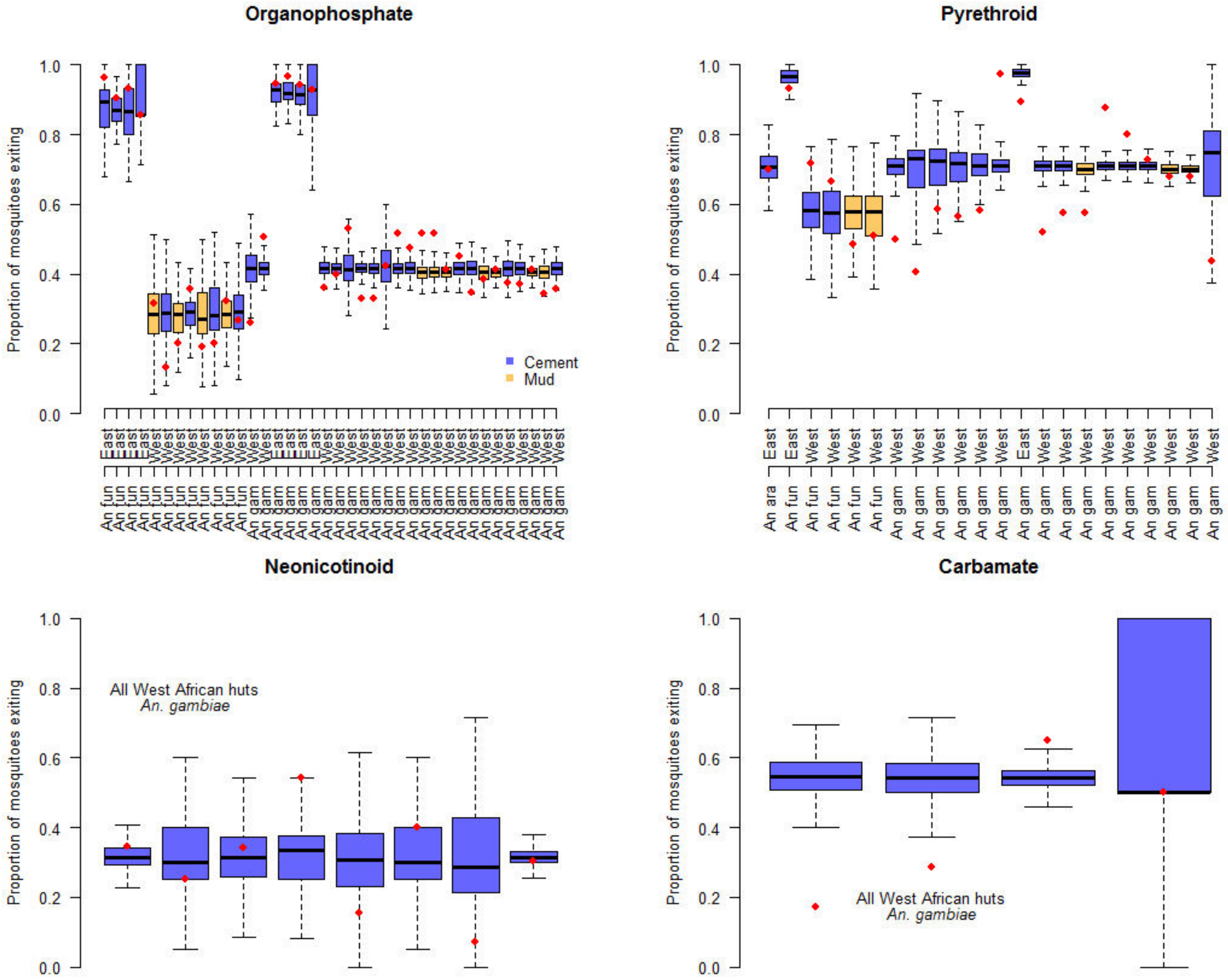


**Supplementary Figure 3: Bayesian model predictions for the proportion of mosquitoes that have exited in the sprayed experimental huts.** Chemistry classes are shown in each panel, model predictions are determined by mosquito species (An ara = *An. arabiensis*; An fun = *An. funestus*; An gam = *An. gambiae*), hut type (East or West African design[54]) and whether the substrate of the hut was made of mud, yellow, or cement (concrete walled-huts are included as cement), in blue. Boxplots show the median (line), 25th and 75th credible intervals (box) and 5th and 95th CrIs for the posterior predictive estimates of the proportion of mosquitoes that exit in the presence of each chemistry. Red points indicate the actual data estimate. The proportion of mosquitoes that exit from spraying in experimental huts is predicted to be greater in the presence of pyrethroids and bendiocarb relative to alternative chemistries, greater in East African huts than West African huts, but relatively similar for different substrates. Note that there are minimal data for neonicotinoids and carbamates, *An. arabiensis* and mud-walled huts.

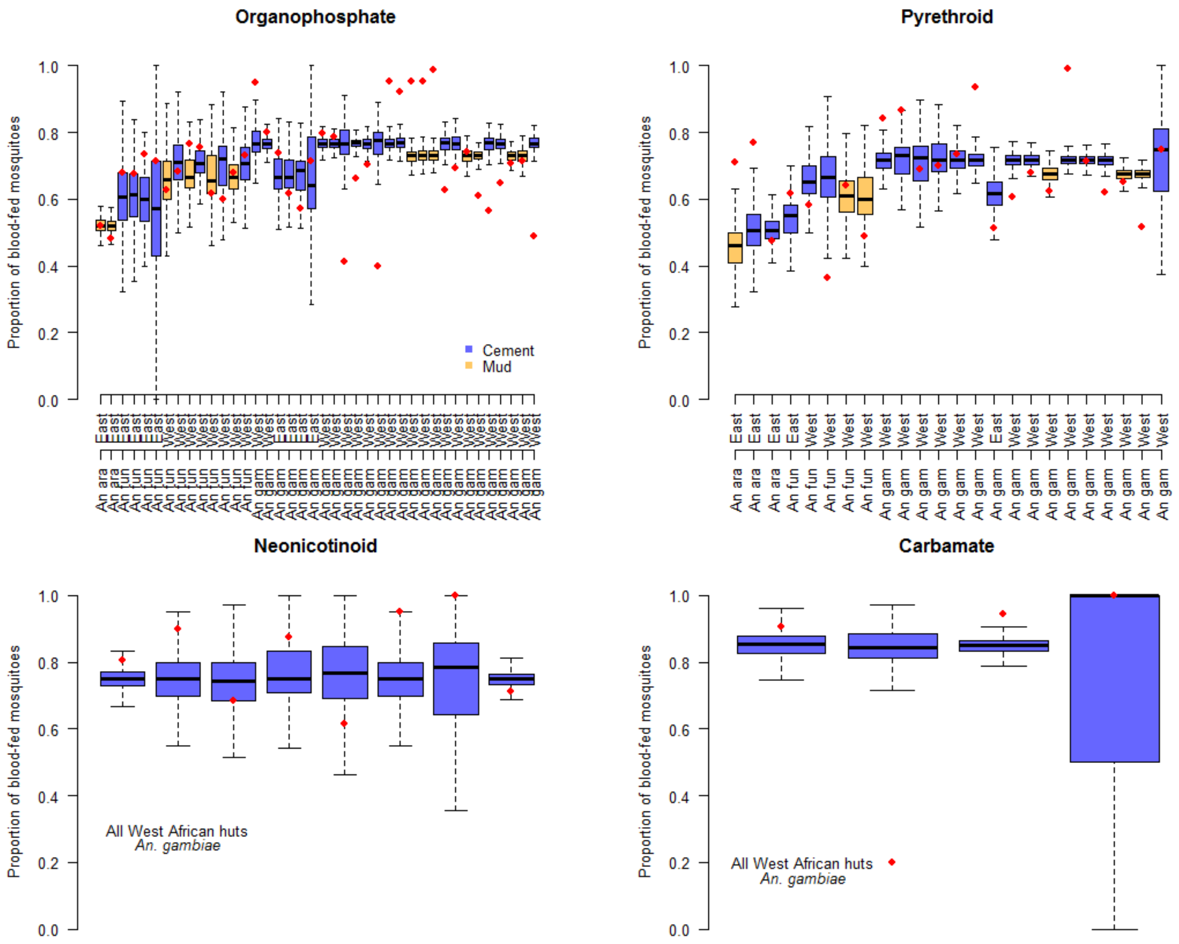


**Supplementary Figure 4: Bayesian model predictions for the proportion of mosquitoes that have blood-fed in the sprayed experimental huts.** Chemistry classes are shown in each panel, model predictions are determined by mosquito species (An ara = *An. arabiensis*; An fun = *An. funestus*; An gam = *An. gambiae*), hut type (East or West African design[54]) and whether the substrate of the hut was made of mud, yellow, or cement (concrete walled-huts are included as cement), in blue. Boxplots show the median (line), 25th and 75th credible intervals (box) and 5th and 95th CrIs for the posterior predictive estimates of the proportion of mosquitoes that are blood-fed by each chemistry. Red points indicate the actual data estimate. The proportion of mosquitoes that blood-fed in sprayed experimental huts is predicted to be broadly comparable across chemistries, reduced in East African huts relative to West African huts and slightly reduced in mud-walled relative to cement-walled huts. Fewer *An. arabiensis* are predicted to have blood-fed relative to other Anophelines but note that there are minimal data for neonicotinoids and carbamates, *An. arabiensis* and mud-walled huts.

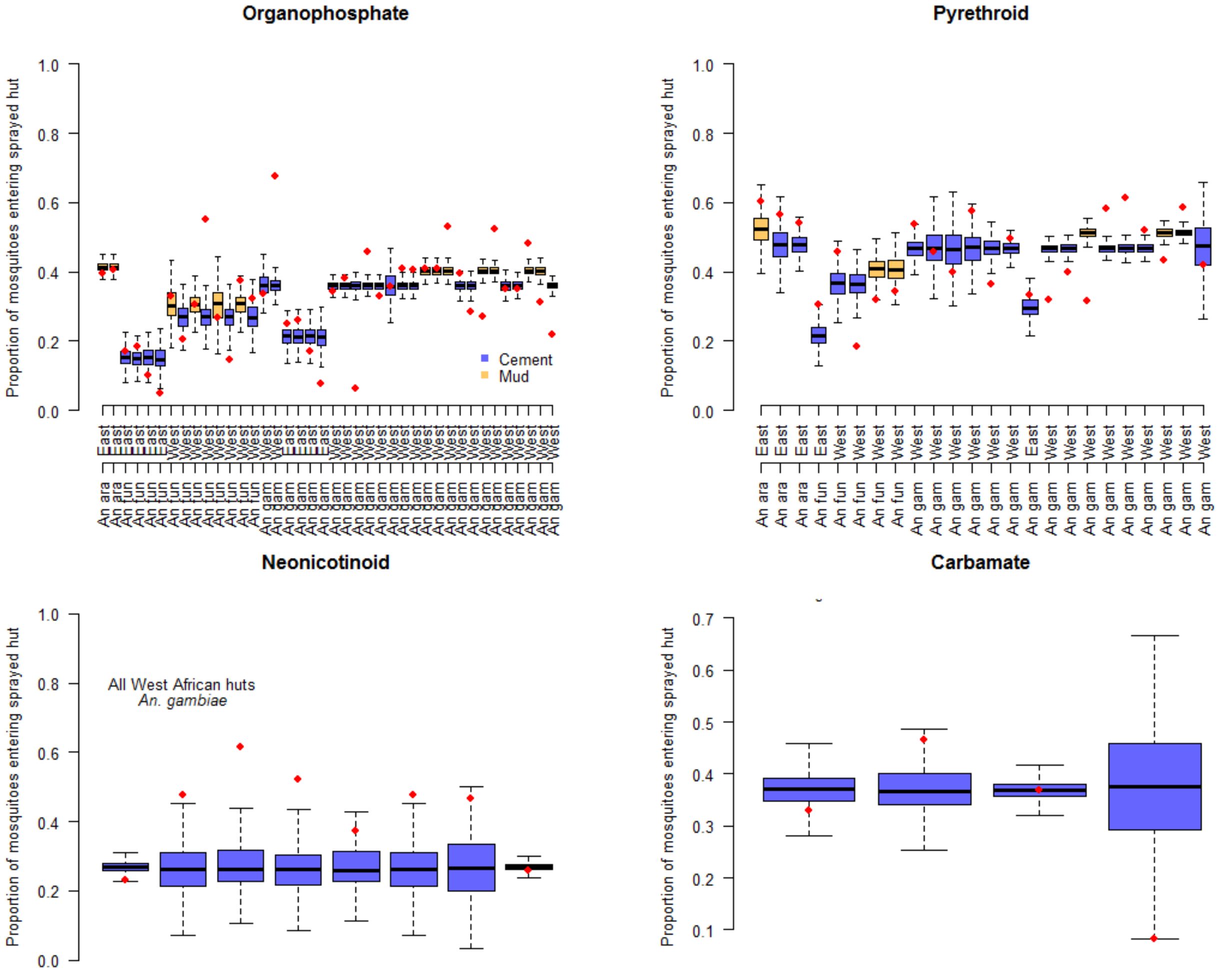


**Supplementary Figure 5: Bayesian model predictions for the proportion of mosquitoes that have entered into sprayed experimental huts out of all mosquitoes entering either sprayed or control huts.** Chemistry classes are shown in each panel, model predictions are determined by mosquito species (An ara = *An. arabiensis*; An fun = *An. funestus*; An gam = *An. gambiae*), hut type (East or West African design[54]) and whether the substrate of the hut was made of mud, yellow, or cement (concrete walled-huts are included as cement), in blue. Boxplots show the median (line), 25$^{th}$ and 75$^{th}$ credible intervals (box) and 5$^{th}$ and 95$^{th}$ CrIs for the posterior predictive estimates of the proportion of mosquitoes that visited the sprayed hut out of all mosquitoes (those entering sprayed and those entering control huts) in the presence of each chemistry. Red points indicate the actual data estimate. The proportion of mosquitoes visiting sprayed experimental huts is predicted to be broadly comparable across chemistries, reduced in East African huts relative to West African huts and slightly reduced in mud-walled relative to cement-walled huts. More *An. arabiensis* are predicted to have visited sprayed huts relative to other Anophelines but note the minimal data for neonicotinoids and carbamates, *An. arabiensis* and mud-walled huts.

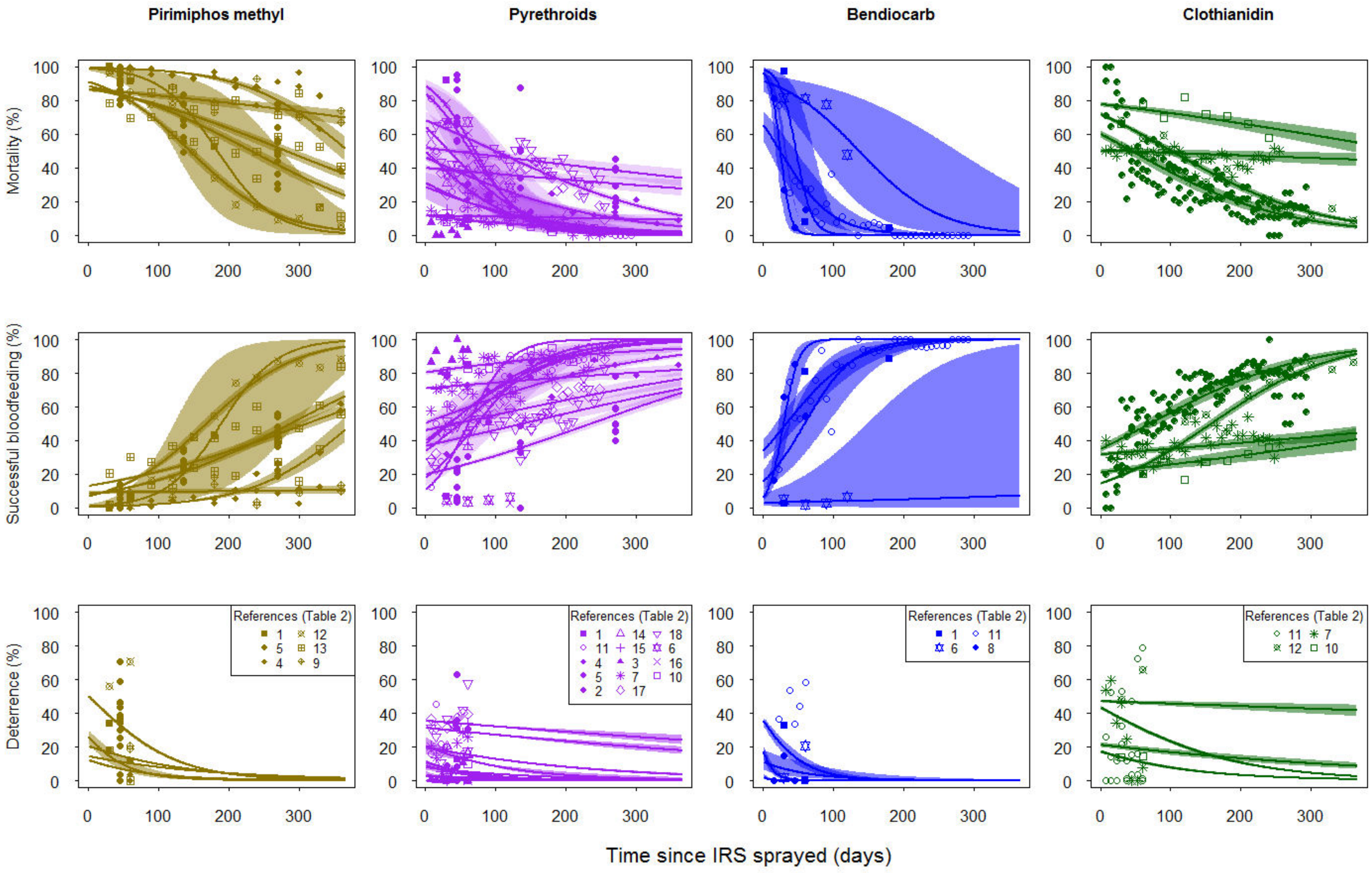

Pirimiphos methyl
Pyrethroids
Bendiocarb
Clothianidin
Mortality (%)
Successful bloodfeeding (%)
Deterrence (%)
References (Table 2)
Time since IRS sprayed (days)

**Supplementary Figure 6:** The probability of mosquitoes dying (top row), successfully blood-feeding (surviving and feeding) (row 2), or being deterred (row 3) are defined for different experimental hut trials over time. Each study is shown independently with 90% credible intervals (shaded region) around the mean best-fit (solid line). The different IRS products; pirimiphos-methyl: Actellic®300CS (column 1), pyrethroids: lambda-cyhalothrin, deltamethrin and alpha-cyhalothrin (column 2), bendiocarb: bendiocarb (1 spray round per year) (column 3) and neonicotinoids: SumiShield®50WG (column 4) are shown and further information on each study are indicated by the symbol shapes (see legend key which refers to study numbers in Table 2 main text).

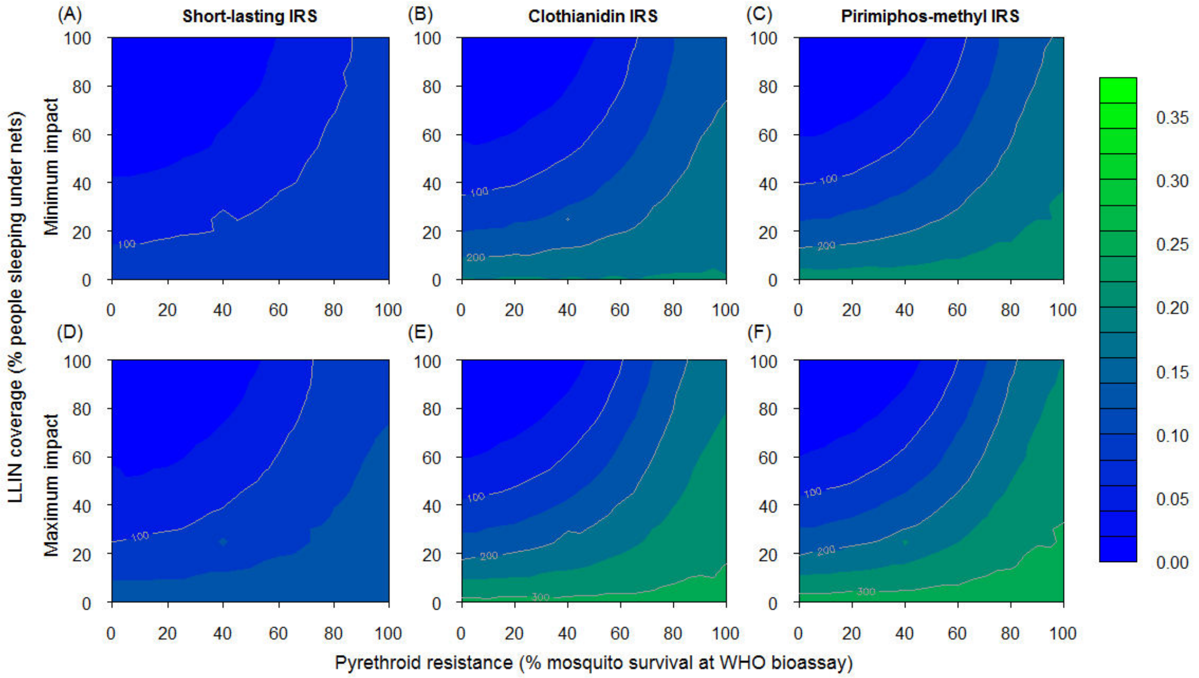


**Supplementary Figure 7: The additional minimal (top row) and maximal (bottom row) impact of adding IRS to bed nets in a perennial setting.** The predicted number of malaria cases averted by annual IRS to a population with an existing level of bednet use (0% to 100% cover, y-axes) and a defined level of pyrethroid resistance (measured as percentage survival in a standard pyrethroid discriminating dose bioassay, x-axes) assuming the worst-case scenario for IRS impact. Clinical cases averted are measured per person per year, following standard LLIN distribution in a moderate endemicity area (30% prevalence in 2 – 10-year olds in the absence of interventions) with perennial transmission (a-c), highly seasonal transmission (d-f). In all panels IRS is applied, untargeted, to 80% of the population using either the short-acting IRS product, Bendiocarb, applied annually (a and d), a long-lasting IRS product (Sumishield®50WG) (b and e) or Actellic®300CS (c and f) . Long-lasting products avert more cases though short-lasting products perform substantially better in highly seasonal settings (Fig. 5 main text).

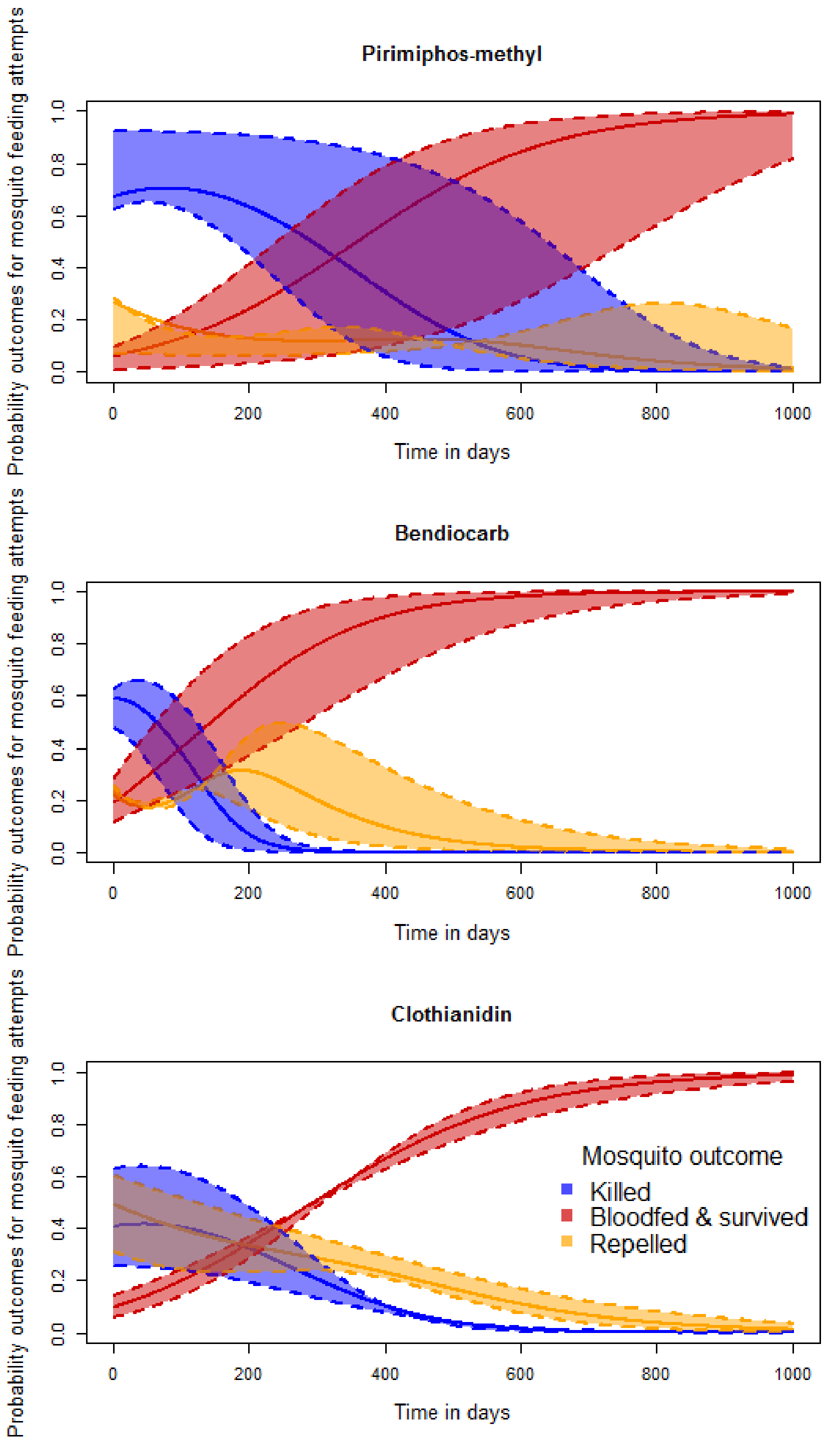


**Supplementary Figure 8: The probabilities that a mosquito entering a sprayed hut successfully blood-feeds, exits without feeding or dies, denoted $s_S$, $r_S$ and $d_S$ respectively (see Supplementary Equations 7-9).** The uncertainty is generated by estimating the best and worst performing experimental hut data for each IRS product (see Supplementary Fig. 6). Estimates are then adjusted as noted in Supplementary Equations 4 – 9. Pyrethroid IRS is handled differently because the impact of resistance is determined adding to the uncertainty for different locations (see Supplementary Fig. 9).

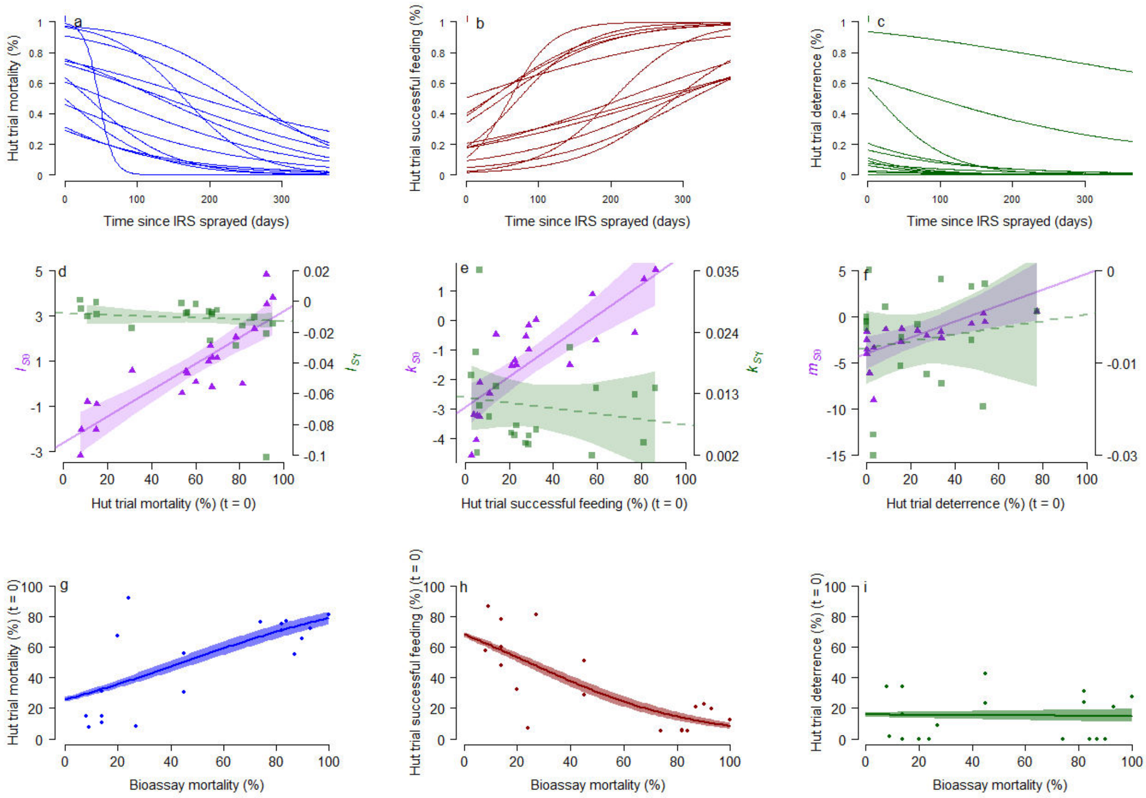


**Supplementary Figure 9: The method used to define the impact of pyrethroid resistance (approximated using the proportion of mosquitoes surviving at a 24-hour susceptibility bioassay test) on the performance of pyrethroid-IRS.** There are 11 datasets with a standard pyrethroid discriminating dose bioassay measurement and initial mosquito mortality estimates measured in experimental huts. There are a further 14 datasets with time series information that have varying initial mosquito mortality (or blood-feeding or deterrence) estimates measured in experimental huts. (a-c) Each of the 14 datasets are fitted separately and defined by the same function as Supplementary Equations 1-3. This provides a vector of estimates for the parameters $l_{S\vartheta}$ , $l_{S\gamma}$ (d); $k_{S\vartheta}$ , $k_{S\gamma}$ (e), and; $m_{S\vartheta}$ , $m_{S\gamma}$ (f) (see Supplementary Equations 14-19) that can be linearly associated with the proportion of mosquitoes that are killed (d), successfully fed (fed and not killed) (e), or deterred (f) at the very start of the IRS experimental hut trial (*t = 0*). The initial mosquito mortality, blood-feeding or deterrence measured in experimental huts can be associated with a standard pyrethroid discriminating dose bioassay for the 11 studies with both data (g-i) as per Supplementary Equations 11-13. Combining these equations enables us to determine the effect of insecticide resistance on the impact of pyrethroid IRS as it changes over time since the pyrethroid active ingredient was first sprayed.

## RStan code for statistical models

## Analysis 1

Are different indoor residual spray (IRS) products more or less effective at causing mosquito mortality, exiting or inhibiting blood-feeding, or causing deterrence, given the local mosquito species, hut type and wall substrate tested in the experimental hut trial?

Data are measured within 2 months of the start of the trial, when huts are first sprayed. This statistical analysis is used to explore the initial impact of IRS products.

Data used here correspond to Supplementary data 1, analysis 1.

```
#######################################
##                                   ##
## PROPORTION OF MOSQUITOES DYING    ##
##                                   ##
#######################################

library(rstan)
rstan_options(auto_write = TRUE)
library(rstanarm)

C = data1$Ntotaldied_IRS
N = data1$Ntotalfemalemosq_IRS
NC = N-C

bglm_1 <- stan_glm(cbind(C, NC) ~ product_type + species_cleaned + Hut + wall_type,
                              data = data1,
                              family = binomial(link = "logit"),
                              chains = 4, cores = 4, seed = 3529075)
bglm_1

#######################################
##                                   ##
## PROPORTION OF MOSQUITOES EXITING ##
##                                   ##
#######################################

data1_exit = data1[complete.cases(data1$Nexittraps_IRS),]

C2 = data1_exit$Nexittraps_IRS
N2 = data1_exit$Ntotalfemalemosq_IRS
NC2 = N2-C2

bglm_2 <- stan_glm(cbind(C2, NC2) ~ product_type + species_cleaned + Hut + wall_type,
                   data = data1_exit,
                   family = binomial(link = "logit"),
                   chains = 4, cores = 4, seed = 3529075)
bglm_2

#######################################
##                                   ##
## PROPORTION OF MOSQUITOES FEEDING ##
##                                   ##
```

```
######################################

C3 = data1$Nbloodfed_IRS
N3 = data1$Ntotalfemalemosq_IRS
NC3 = N3-C3

bglm_3 <- stan_glm(cbind(C3, NC3) ~ product_type + species_cleaned + Hut + wall_type,
                   data = data1,
                   family = binomial(link = "logit"),
                   chains = 4, cores = 4, seed = 3529075)
bglm_3

######################################
##                                  ##
## PROPORTION OF MOSQUITOES VISITING##
##                                  ##
######################################

C4 = data1$Ntotalfemalemosq_IRS
N4 = data1$Ntotalfemalemosq_IRS + data1$Ntotalfemalemosq_C
NC4 = data1$Ntotalfemalemosq_C

bglm_4 <- stan_glm(cbind(C4, NC4) ~ product_type + species_cleaned + Hut + wall_type,
                   data = data1,
                   family = binomial(link = "logit"),
                   chains = 4, cores = 4, seed = 3529075)
bglm_4
```

## Analysis 2

How does the function of each IRS product change over time?

Data used here correspond to Supplementary data 1, analysis 2.

```
##Data preparation

IRS_cleaner_f_base = function(data1){

  n_t=data1$Ntotalfemalemosq_IRS
  d_t=data1$Ntotaldied_IRS
  f_t=round(data1$Nbloodfed_IRS * (1 - data1$Ntotaldied_IRS/data1$Ntotalfemalemosq_IRS),0)
  deterrence_IRS = ifelse(c(data1$Ntotalfemalemosq_C-data1$Ntotalfemalemosq_IRS)<0,0,
                          c(data1$Ntotalfemalemosq_C-data1$Ntotalfemalemosq_IRS))
  deterrence_total = c(data1$Ntotalfemalemosq_IRS+data1$Ntotalfemalemosq_C)
  time=data1$Months_since_IRS*30

  return(list(N=nrow(data1),
              n_t=n_t,
              d_t=d_t,
              fed_t=f_t,
              deterrence_IRS = deterrence_IRS,
              deterrence_total = deterrence_total,
              time=time) )
```

```
}

// Binomial logistic function
// Statistical analysis of changing mosquito behaviour over time
data {

  int<lower=1> N;                  // Numnber of rows of data

  int<lower=0> n_t[N];             // Total number of mosquitoes entering
  int<lower=0> d_t[N];             // Number mosquites dead
  int<lower=0> f_t[N];           // Number of mosquitoes feeding

  int<lower=0> deterrence_IRS[N];  // Number of mosquitoes in sprayed huts
  int<lower=0> deterrence_total[N];// Total number of mosquitoes in all huts

  vector<lower=0>[N] time;         // predictor time

}

parameters {
  //Consider mortality. Proportion of mosquitoes dying
  real alpha1;
  real alpha2;

  //Consider feeding. Proportion of mosquitoes that successfully fed
  real beta1;
  real beta2;

  //Consider feeding. Proportion of mosquitoes in treatment hut
  real omega1;
  real omega2;

  //  vector[N_study] study_a;
  //  real<lower=0,upper=10> sigma;
}

model {
  real sp[N];
  real fp[N];
  real det[N];

  alpha1 ~ normal(0,100);
  alpha2 ~ normal(0,100);

  beta1 ~ normal(0,100);
  beta2 ~ normal(0,100);

  omega1 ~ normal(0,100);
  omega2 ~ normal(0,100);

  //  study_a ~ normal(0,sigma);

  for (n in 1:N) {
```

```
    sp[n] = alpha1  + alpha2 * time[n];
    fp[n] = beta1  + beta2 * time[n];
    det[n] = omega1  + omega2 * time[n];
  }

  d_t ~ binomial_logit(n_t, sp);
  f_t ~ binomial_logit(n_t, fp);
  deterrence_IRS ~ binomial_logit(deterrence_total, det);
}
```

Modifying this code allows us to fit to each individual study:

```
data {

  int<lower=1> N;                    // Numnber of rows of data

  int<lower=0> n_t[N];               // Total number of mosquitoes entering
  int<lower=0> d_t[N];               // Number mosquites dead
  int<lower=0> fed_t[N];             // Number of mosquitoes feeding

  int<lower=0> deterrence_IRS[N];  // Number of mosquitoes in sprayed huts
  int<lower=0> deterrence_total[N];// Total number of mosquitoes in all huts

  vector<lower=0>[N] time;           // predictor time

  int<lower=1> N_IRS;                // Maximum number of individual IRS data
  int<lower=1, upper=N_IRS> IRS[N];// Numerical identification of these individual data

}

parameters {

  real alpha1[N_IRS];
  real alpha2[N_IRS];

  real beta1[N_IRS];
  real beta2[N_IRS];

  real omega1[N_IRS];
  real omega2[N_IRS];

}

model {
  real sp[N];
  real fp[N];
  real det[N];

  alpha1 ~ normal(0,10);
  alpha2 ~ normal(0,10);

  beta1 ~ normal(0,10);
  beta2 ~ normal(0,10);
```

```
  omega1 ~ normal(0,10);
  omega2 ~ normal(0,10);

  for (n in 1:N) {
    sp[n] = alpha1[IRS[n]] + alpha2[IRS[n]] * time[n];
    fp[n] = beta1[IRS[n]]  + beta2[IRS[n]]  * time[n];
    det[n] = omega1[IRS[n]]  + omega2[IRS[n]]  * time[n];
  }

  d_t ~ binomial_logit(n_t, sp);
  fed_t ~ binomial_logit(n_t, fp);
  deterrence_IRS ~ binomial_logit(deterrence_total, det);
}

generated quantities{
  real sp_ppc[N_IRS, 365];
  real fp_ppc[N_IRS, 365];
  real det_ppc[N_IRS, 365];


  for(v in 1:N_IRS){
    for(t in 1:365){
      sp_ppc[v, t] = binomial_rng(365, inv_logit(alpha1[v] + alpha2[v] * t)) / 365.0;
      fp_ppc[v, t] = binomial_rng(365, inv_logit(beta1[v] + beta2[v] * t)) / 365.0;
      det_ppc[v, t] = binomial_rng(365, inv_logit(omega1[v] + 0 * t)) / 365.0;
    }
  }
}
```

## Analysis 3a

What is the relationship between:

- WHO bioassay mortality and 24-hour mortality,
- WHO bioassay mortality and successful 24-hour blood feeding,
- WHO bioassay mortality and 24-hour deterrence,
  - in experimental huts at the very start of the IRS trial (t = 1 day)?

Data used here correspond to Supplementary data 1, analysis 3a.

```
// bernoulli_logistic transformed data function
data {

  int<lower=1> N;                // rows of data

  int<lower=0> d_t[N];           // Number of mosquitoes dying IRS HUTS
  int<lower=0> n_t[N];           // Total number of mosquitoes in IRS huts
  int<lower=0> f_t[N];           // Number of mosquitoes feeding in HUTS

  int<lower=0> n_det[N];       // Number deterred by spray
  int<lower=0> n_c[N];         // Denomimator for deterrence
  vector<lower=0>[N] x;        // predictor bioassay mortality

  int<lower=1> N_IRS;            // IRS treatments
```

```
  int<lower=1, upper=N_IRS> IRS[N];

  }

parameters {

  real alpha1_tilde[N_IRS];
  real mu_alpha1;
  real sigma_alpha1;

  real alpha2_tilde[N_IRS];
  real mu_alpha2;
  real sigma_alpha2;

  real beta1_tilde[N_IRS];
  real mu_beta1;  //
  real sigma_beta1;//

  real beta2_tilde[N_IRS];
  real mu_beta2;  //
  real sigma_beta2;//

  real theta2_tilde[N_IRS];
  real mu_theta2;  //
  real sigma_theta2;//

  real theta1_tilde[N_IRS];
  real mu_theta1;  //
  real sigma_theta1;//


}

transformed parameters {
    real alpha1[N_IRS];
    real alpha2[N_IRS];

    real beta1[N_IRS];
    real beta2[N_IRS];

    real theta1[N_IRS];
    real theta2[N_IRS];

  for (v in 1:N_IRS) {
    alpha1[v] = mu_alpha1 + sigma_alpha1 * alpha1_tilde[v];
    alpha2[v] = mu_alpha2 + sigma_alpha2 * alpha2_tilde[v];

    beta1[v] = mu_beta1 + sigma_beta1 * beta1_tilde[v];
    beta2[v] = mu_beta2 + sigma_beta2 * beta2_tilde[v];

    theta1[v] = mu_theta1 + sigma_theta1 * theta1_tilde[v];
    theta2[v] = mu_theta2 + sigma_theta2 * theta2_tilde[v];
```

```
  }
}

model {
  real y_tilde[N];
  real k_tilde[N];
  real det_tilde[N];

  for(n in 1:N)
    y_tilde[n] = alpha2[IRS[n]] * x[n] + alpha1[IRS[n]];

  alpha1_tilde ~ normal(0, 1);
  mu_alpha1 ~ normal(0, 10);
  sigma_alpha1 ~ normal(0, 5);


  alpha2_tilde ~ normal(0, 1);
  mu_alpha2 ~ normal(0, 10);
  sigma_alpha2 ~ normal(0, 5);

  d_t ~ binomial_logit(n_t, y_tilde);


  for(n in 1:N)
    k_tilde[n] = beta2[IRS[n]] * x[n] + beta1[IRS[n]];

  beta2_tilde ~ normal(0, 1);
  mu_beta2 ~ normal(0, 10);
  sigma_beta2 ~ normal(0, 5);

  beta1_tilde ~ normal(0, 1);
  mu_beta1 ~ normal(0, 10);
  sigma_beta1 ~ normal(0, 5);

  f_t ~ binomial_logit(n_t, k_tilde);


 for(n in 1:N)
    det_tilde[n] = theta2[IRS[n]] * x[n] + theta1[IRS[n]];

  theta2_tilde ~ normal(0, 1);
  mu_theta2 ~ normal(0, 10);
  sigma_theta2 ~ normal(0, 5);

  theta1_tilde ~ normal(0, 1);
  mu_theta1 ~ normal(0, 10);
  sigma_theta1 ~ normal(0, 5);

  n_det ~ binomial_logit(n_c, det_tilde);

  }

generated quantities {
```

```
  real y_ppc[N_IRS, 100];
  real k_ppc[N_IRS, 100];
  real det_ppc[N_IRS, 100];

  for(v in 1:N_IRS) {
    for (t in 1:100) {
      y_ppc[v, t] = binomial_rng(100, inv_logit(alpha2[v] * t + alpha1[v])) / 100.0;
      k_ppc[v, t] = binomial_rng(100, inv_logit(beta2[v] * t + beta1[v])) / 100.0;
      det_ppc[v, t] = binomial_rng(100, inv_logit(theta2[v] * t + theta1[v])) / 100.0;
    }
  }
}

## Model parameter predictions

alpha1 =   -1.059923  ## mean
alpha2 =    0.02387883
beta1  =    0.7674771
beta2  =   -0.03166185
theta1 =   -1.673563
theta2 =   -0.0007511497

alpha1qu = -1.1387719 ## upper 90% credible interval
alpha2qu =  0.02226354
beta1qu  =  0.6885264
beta2qu  = -0.03346408
theta1qu =- 1.794143
theta2qu = -0.003069259

alpha1ql = -0.9785583 ## lower 90% credible interval
alpha2ql =  0.02556298
beta1ql =   0.8468829
beta2ql =  -0.02982156
theta1ql = -1.566623
theta2ql =  0.001473022

x_bioassay_mort = rev(seq(0,100,length = 101))
```

## Analysis 3b

The individual studies for pyrethroid IRS have varying impacts on mosquito mortality, blood-feeding and deterrence, We estimate parameters for each of these relationships for each study. These can be associated with the estimated mortality, blood-feeding or deterrence at time t = 1 day. As the association between mortality, blood-feeding and deterrence at time t = 1 and WHO bioassay mortality is defined in analysis 3a, it is then possible to determine the relationship between WHO biassay mortality and pyrethroid products over time. Supporting methods 2 provides details of this association.

Data used here correspond to Supplementary data 1, analysis 3b.

```
// bernoulli_logistic transformed data function
data {
  int<lower=1> N;                   // rows of data
  int<lower=0> n_t[N];              // Total number of mosquitoes entering IRS huts
```

```
  int<lower=0> d_t[N];             // Number mosquites dead sprayed hut
  int<lower=0> f_t[N];           // Number of mosquitoes feeding

  int<lower=0> deterrence_IRS[N];  // Number of mosquitoes in sprayed huts
  int<lower=0> deterrence_total[N]; //Total number of mosquitoes iall huts
  vector<lower=0>[N] time;       // predictor
  int<lower=1> N_IRS;           // IRS treatments
  int<lower=1, upper=N_IRS> IRS[N];
}

parameters {
  real alpha1x[N_IRS];
  real alpha2x[N_IRS];

  real beta1x[N_IRS];
  real beta2x[N_IRS];

  real omega1x[N_IRS];
  real omega2x[N_IRS];
}

model {
  real sp[N];
  real fp[N];
  real det[N];

  alpha1x ~ normal(0,10);
  alpha2x ~ normal(0,10);

  beta1x ~ normal(0,10);
  beta2x ~ normal(0,10);

  omega1x ~ normal(0,10);
  omega2x ~ normal(0,10);

  for (n in 1:N) {
    sp[n] = alpha1x[IRS[n]] + alpha2x[IRS[n]] * time[n];
    fp[n] = beta1x[IRS[n]]  + beta2x[IRS[n]]  * time[n];
    det[n] = omega1x[IRS[n]]  + omega2x[IRS[n]]  * time[n];
  }



  d_t ~ binomial_logit(n_t, sp);
  f_t ~ binomial_logit(n_t, fp);
  deterrence_IRS ~ binomial_logit(deterrence_total, det);
}

generated quantities{
  real sp_ppc[N_IRS, 365];
  real fp_ppc[N_IRS, 365];
  real det_ppc[N_IRS, 365];
```

```
  for(v in 1:N_IRS){
    for(t in 1:365){
      sp_ppc[v, t] = binomial_rng(365, inv_logit(alpha1x[v] + alpha2x[v] * t)) / 365.0;
      fp_ppc[v, t] = binomial_rng(365, inv_logit(beta1x[v] + beta2x[v] * t)) / 365.0;
      det_ppc[v, t] = binomial_rng(365, inv_logit(omega1x[v] + alpha2x[v] * t)) / 365.0;
    }
  }
}
```

Using linear associations, this gives us the following intercept and gradient for each parameter association to the respective impact

- *mort1* = hut mortality at time t = 1 day
- *succ1* = hut feeding success at time t = 1 day
- *deterrence* = deterrence from huts at time t = 1 day
  - alpha1x ~ grad1 x *mort1* + int1
  - alpha2x ~ grad2 x *mort2* + int2
  - beta1x ~ grad3 x *succ1* + int3
  - beta2x ~ grad4 x *succ2* + int4
  - omega1x ~ grad5 x *deterrence* + int5
  - alpha2x ~ grad6 x *deterrence* + int6
- *(We assume the same depreciation in deterrence as that for mortality)*

```
int1 =  -2.587528
int2 =  -0.002989876
int3 =  -2.95455
int4 =   0.01200367
int5 =  -3.917539
int6 =   0.002780567

grad1 =  5.777369
grad2 = -0.01362388
grad3 =  5.231271
grad4 = -0.004870386
grad5 =  8.542869
grad6 =  0.005316776
```

Using the above we can estimate the parameters needed to define the predicted temporal impact of pyrethroid IRS at any level of resistance to pyrethroids (as determined by WHO discriminatory dose bioassay tests).

```
params = rev(seq(0,1,length=21))
vals_vec = rev(seq(1,101,length=21))

params_store = array(dim=c(length(params),7))
params_store[,1] = params

for(i in 1:length(params)){
  temp_mort_1 = grad1 * (1 / (1 + exp(-alpha1 - alpha2 * vals_vec[i]))) + int1
  temp_mort_2 = grad2 * (1 / (1 + exp(-alpha1 - alpha2 * vals_vec[i]))) + int2

  temp_succ_1 = grad3 * (1 / (1 + exp(-beta1 - beta2 * vals_vec[i]))) + int3
  temp_succ_2 = grad4 * (1 / (1 + exp(-beta1 - beta2 * vals_vec[i]))) + int4

```

```
  temp_det_1 = grad5 * (1 / (1 + exp(-theta1 - theta2 * vals_vec[i]))) + int5
  temp_det_2 = grad6 * (1 / (1 + exp(-theta1 - theta2 * vals_vec[i]))) + int6

  params_store[i,2] = temp_mort_1
  params_store[i,3] = temp_mort_2
  params_store[i,4] = temp_succ_1
  params_store[i,5] = temp_succ_2
  params_store[i,6] = temp_det_1
  params_store[i,7] = temp_mort_2

}
colnames(params_store) = c("Prop_dying_at_bioassay",
                           "irs_decay_mort1_1", "irs_decay_mort2_1",
                           "irs_decay_succ1_1", "irs_decay_succ2_1",
                           "irs_decay_det1_1",  "irs_decay_det2_1")
```

Parameter estimates for the analysis are provided in Supplementary data table S3.

**References**

1. Akogbéto, M. C., Padonou, G. G., Gbénou, D., Irish, S. & Yadouleton, A. Bendiocarb, a potential alternative against pyrethroid resistant Anopheles gambiae in Benin, West Africa. *Malar. J.* **9,** 204 (2010).

2. Yadouleton, A. W. *et al.* Insecticide resistance status in Anopheles gambiae in southern Benin. *Malar. J.* **9,** 83 (2010).

3. Agossa, F. R. *et al.* Efficacy of various insecticides recommended for indoor residual spraying: pirimiphos methyl, potential alternative to bendiocarb for pyrethroid resistance management in Benin, West Africa. *Trans. R. Soc. Trop. Med. Hyg.* **108,** 84–91 (2014).

4. Agossa, F. R., Gnanguenon, V., Anagonou, R. & Azondekon, R. Impact of Insecticide Resistance on the Effectiveness of Pyrethroid-Based Malaria Vectors Control Tools in Benin : Decreased Toxicity and Repellent Effect. *PLoS One* **10,** 1–13 (2015).

5. Agossa, F. R. *et al.* Efficacy of a novel mode of action of an indoor residual spraying product, SumiShield® 50WG against susceptible and resistant populations of Anopheles gambiae (s.l.) in Benin, West Africa. *Parasit. Vectors* **11,** 293 (2018).

6. Asale, A. *et al.* Evaluation of the efficacy of DDT indoor residual spraying and long-lasting insecticidal nets against insecticide resistant populations of Anopheles arabiensis Patton (Diptera: Culicidae) from Ethiopia using experimental huts. *Parasit. Vectors* **7,** 131 (2014).

7. Barasa, S. Evaluation of existing spatial repellents for the control of malaria vectors in rural Tanzania SHEILA OGOMA BARASA BSc , MSc . Thesis submitted in accordance with the requirements for the degree of Doctor of Philosophy of the University of London MARCH 20. (2015).

8. Chandre, F. *et al.* Field efficacy of pyrethroid treated plastic sheeting (durable lining) in combination with long lasting insecticidal nets against malaria vectors. *Parasit. Vectors* **3,** 65 (2010).
9. Diabate, A. *et al.* The indoor use of plastic sheeting pre-impregnated with insecticide for control of malaria vectors. *Trop. Med. Int. Heal.* **11,** 597–603 (2006).
10. Djènontin, A. *et al.* Indoor use of plastic sheeting impregnated with carbamate combined with long-lasting insecticidal mosquito nets for the control of pyrethroid-resistant malaria vectors. *Am. J. Trop. Med. Hyg.* **83,** 266–270 (2010).
11. Kitau, J. *et al.* Indoor residual spraying with microencapsulated DEET repellent ( N , N-diethyl-m-toluamide ) for control of Anopheles arabiensis and Culex quinquefasciatus. 1–11 (2014). doi:10.1186/1756-3305-7-446
12. Malima, R. *et al.* Experimental hut evaluation of a novel long-lasting non-pyrethroid durable wall lining for control of pyrethroid-resistant Anopheles gambiae and Anopheles funestus in Tanzania. *Malar. J.* **16,** 82 (2017).
13. Mosqueira, B. *et al.* Efficacy of an insecticide paint against malaria vectors and nuisance in West Africa - Part 2: Field evaluation. *Malar. J.* **9,** 341 (2010).
14. Mosqueira, B. *et al.* Proposed use of spatial mortality assessments as part of the pesticide evaluation scheme for vector control. *Malar. J.* **12,** 366 (2013).
15. N'Guessan, R., Rowland, M., Moumouni, T.-L., Kesse, N. B. & Carnevale, P. Evaluation of synthetic repellents on mosquito nets in experimental huts against insecticide-resistant Anopheles gambiae and Culex quinquefasciatus mosquitoes. *Trans. R. Soc. Trop. Med. Hyg.* **100,** 1091–1097 (2006).
16. N'Guessan, R. *et al.* Control of pyrethroid-resistant Anopheles gambiae and Culex quinquefasciatus mosquitoes with chlorfenapyr in Benin. *Trop. Med. Int. Heal.* **14,**

389–395 (2009).

17. N’guessan, R. *et al.* Control of pyrethroid and DDT-resistant Anopheles gambiae by application of indoor residual spraying or mosquito nets treated with a long-lasting organophosphate insecticide, chlorpyrifos-methyl. *Malar. J.* **9,** 44 (2010).

18. N’Guessan, R., Corbel, V., Akogbéto, M. & Rowland, M. Reduced efficacy of insecticide-treated nets and indoor residual spraying for malaria control in pyrethroid resistance area, Benin. *Emerg. Infect. Dis.* **13,** 199–206 (2007).

19. Corbel, V. *et al.* Multiple insecticide resistance mechanisms in Anopheles gambiae and Culex quinquefasciatus from Benin, West Africa. *Acta Trop.* **101,** 207–216 (2007).

20. Ngufor, C. *et al.* Chlorfenapyr (A Pyrrole Insecticide) Applied Alone or as a Mixture with Alpha-Cypermethrin for Indoor Residual Spraying against Pyrethroid Resistant Anopheles gambiae sl: An Experimental Hut Study in Cove, Benin. *PLoS One* **11,** e0162210 (2016).

21. Ngufor, C. *et al.* Which intervention is better for malaria vector control: insecticide mixture long-lasting insecticidal nets or standard pyrethroid nets combined with indoor residual spraying? *Malar J* **16,** 340 (2017).

22. Ngufor, C., Fongnikin, A., Rowland, M. & N’Guessan, R. Indoor residual spraying with a mixture of clothianidin (a neonicotinoid insecticide) and deltamethrin provides improved control and long residual activity against pyrethroid resistant Anopheles gambiae sl in Southern Benin. *PLoS One* **12,** e0189575 (2017).

23. Ngufor, C. *et al.* Combining indoor residual spraying with chlorfenapyr and long-lasting insecticidal bed nets for improved control of pyrethroid-resistant Anopheles gambiae: an experimental hut trial in Benin. *Malar. J.* **10,** 343 (2011).

24. Ngufor, C. *et al.* Combining organophosphate treated wall linings and long-lasting

insecticidal nets for improved control of pyrethroid resistant Anopheles gambiae. *PLoS One* **9,** e83897 (2014).

25. Ngufor, C. *et al.* Combining organophosphate-treated wall linings and long-lasting insecticidal nets fails to provide additional control over long-lasting insecticidal nets alone against multiple insecticide-resistant Anopheles gambiae in Cote d'Ivoire: an experimental hut. *Malar J* **13,** 396 (2014).

26. Ogoma, S. B. *et al.* An experimental hut study to quantify the effect of DDT and airborne pyrethroids on entomological parameters of malaria transmission. *Malar. J.* **13,** 131 (2014).

27. Okumu, F. O. *et al.* Comparative field evaluation of combinations of long-lasting insecticide treated nets and indoor residual spraying, relative to either method alone, for malaria prevention in an area where the main vector is Anopheles arabiensis. *Parasit. Vectors* **6,** 46 (2013).

28. Okumu, F. O. & Moore, S. J. *Final Scientific Report for PMI Activity TZ-1,2: IRS and LLIN: Integration of methods and insecticide mode of actions for control of African malaria vector mosquitoes 5 th September 2012*.

29. Okumu, F. O. *et al.* A Modified Experimental Hut Design for Studying Responses of Disease-Transmitting Mosquitoes to Indoor Interventions: The Ifakara Experimental Huts. *PLoS One* **7,** e30967 (2012).

30. Oxborough, R. M. *et al.* Evaluation of indoor residual spraying with the pyrrole insecticide chlorfenapyr against pyrethroid-susceptible Anopheles arabiensis and pyrethroid-resistant Culex quinquefasciatus mosquitoes. *Trans. R. Soc. Trop. Med. Hyg.* **104,** 639–645 (2010).

31. Matowo, J. *et al.* Biochemical basis of permethrin resistance in Anopheles arabiensis

from Lower Moshi, north-eastern Tanzania. *Malar. J.* **9,** 193 (2010).

32. Oxborough, R. M. *et al.* Long-lasting control of Anopheles arabiensis by a single spray application of micro-encapsulated pirimiphos-methyl (Actellic® 300 CS). *Malar. J.* **13,** 37 (2014).

33. Oxborough, R. M., Kitau, J., Jones, R., Mosha, F. W. & Rowland, M. W. Experimental hut and bioassay evaluation of the residual activity of a polymer-enhanced suspension concentrate (SC-PE) formulation of deltamethrin for IRS use in the control of Anopheles arabiensis. *Parasit. Vectors* **7,** 454 (2014).

34. Randriamaherijaona, S., Nepomichene, T. N. J. J., Assoukpa, J., Madec, Y. & Boyer, S. Efficacy of Bendiocarb Used for Indoor Residual Spraying for Malaria Control in Madagascar: Results With Local Anopheles Species (Diptera: Culicidae) From Experimental Hut Trials. *J. Med. Entomol.* **54,** 1031–1036 (2017).

35. Randriamaherijaona, S., Nepomichene, T., Assoukpa, J., Madec, Y. & Boyer, S. First evaluation of bendiocarb in experimental huts using different substrates in Madagascar. *Malar. J.* **15,** 293 (2016).

36. Rowland, M. *et al.* A new long-lasting indoor residual formulation of the organophosphate insecticide pirimiphos methyl for prolonged control of pyrethroid-resistant mosquitoes: an experimental hut trial in Benin. *PLoS One* **8,** e69516 (2013).

37. Tchicaya, E. S. *et al.* Micro-encapsulated pirimiphos-methyl shows high insecticidal efficacy and long residual activity against pyrethroid-resistant malaria vectors in central Côte d'Ivoire. *Malar. J.* **13,** 332 (2014).

38. World Health Organization. Dept. of Communicable Disease Prevention Control and Eradication. & WHO Pesticide Evaluation Scheme. Report of the sixth WHOPES working group meeting : WHO/HQ, Geneva, 6-7 November 2002 : review of :

Deltamethrin 25% WG & WP, Agnique MMF. 56 p. (2002).

39. World Health Organization. *Report of the sixteenth WHOPES working group meeting*. (2013).

40. World Health Organization. *Global plan for insecticide resistance management in malaria vectors*. *World Health Organisation* (World Health Organization, 2012).

41. Yeebiyo, Y. *et al.* Short persistence of bendiocarb sprayed on pervious walls and its implication for the indoor residual spray program in Ethiopia. *Parasit. Vectors* **9,** 266 (2016).

42. Griffin, J. T. *et al.* Reducing Plasmodium falciparum malaria transmission in Africa: a model-based evaluation of intervention strategies. *PLoS Med.* **7,** e1000324 (2010).

43. White, M. T. *et al.* Modelling the impact of vector control interventions on Anopheles gambiae population dynamics. *Parasit. Vectors* **4,** 153 (2011).

44. Griffin, J. T., Ferguson, N. M. & Ghani, A. C. Estimates of the changing age-burden of Plasmodium falciparum malaria disease in sub-Saharan Africa. *Nat. Commun.* **5,** 1–10 (2014).

45. Griffin, J. T. *et al.* Gradual acquisition of immunity to severe malaria with increasing exposure. *Proc. R. Soc. B Biol. Sci.* **282,** 20142657–20142657 (2015).

46. Papaspiliopoulos, O., Roberts, G. O. & Sköld, M. A general framework for the parameterization of hierarchical models. *Stat. Sci.* **22,** 59–73 (2007).

47. Betancourt, M. & Girolami, M. in *Current Trends in Bayesian Methodology with Applications* (eds. Upadhyay, S. K., Singh, U., Dey, D. K. & Loganathan, A.) 79–102 (Chapman and Hall CRC Press Taylor & Francis Group, 2015).

48. Stan Development Team. *shinystan: Interactive visual and numerical diagnostics and posterior analysis for Bayesian models*. (2017).

49. Akogbéto, M. C., Padonou, G. G., Gbénou, D., Irish, S. & Yadouleton, A. Bendiocarb, a potential alternative against pyrethroid resistant Anopheles gambiae in Benin, West Africa. *Malar. J.* **9,** 204 (2010).

50. Churcher, T. S., Lissenden, N., Griffin, J. T., Worrall, E. & Ranson, H. The impact of pyrethroid resistance on the efficacy and effectiveness of bednets for malaria control in Africa. *Elife* **5,** (2016).

51. Bagi, J. *et al.* When a discriminating dose assay is not enough: measuring the intensity of insecticide resistance in malaria vectors. *Malar. J.* **14,** 210 (2015).

52. Briët, O. J. *et al.* Effects of pyrethroid resistance on the cost effectiveness of a mass distribution of long-lasting insecticidal nets: a modelling study. *Malar. J.* **12,** 77 (2013).

53. Winskill, P., Walker, P. G., Griffin, J. T. & Ghani, A. C. Modelling the cost-effectiveness of introducing the RTS,S malaria vaccine relative to scaling up other malaria interventions in sub-Saharan Africa. *BMJ Glob. Heal.* **2,** (2017).

54. World Health Organization. *Guidelines for testing mosquito adulticides for indoor residual spraying and treatment of mosquito nets*. (2006).
