# Supplementary material for: Systematic review of indoor residual spray efficacy and effectiveness against Plasmodium falciparum in Africa
Source: Nat Commun. 2018 Nov 26;9:4982. doi: 10.1038/s41467-018-07357-w (PMC6255894; doi:10.1038/s41467-018-07357-w)
Supplement: Supplementary file 3 — Description of Additional Supplementary Files [file 41467_2018_7357_MOESM3_ESM.pdf]

Description of Additional Supplementary Files

File Name: Supplementary Data 1
Description: Supplementary Data 1 gives the raw data used in all analyses with the exception of unpublished data which we note can be requested from the data owners (who are also authors on the manuscript). The file contains a list of the studies included in the metadata and is broken into 4 tabs for clarity.

Analysis 1: The initial impact of IRS products

Analysis 2: Temporal effects of IRS products

Analysis 3a: Pyrethroid resistance, the association between bioassay and experimental hut mortality at the start of an IRS experimental hut trial.

Analysis 3b: Pyrethroid resistance, the interaction between reduced initial efficacy of pyrethroid IRS and the depreciation of this effect over time as pyrethroid resistance increase.

File Name: Supplementary Data 2
Description: Supplementary Data 2 gives the model predictions of the public health benefit of indoor residual spraying at different levels of pyrethroid resistance for each class of insecticide assessed. The file contains model predictions for the mean, minimal and maximal (to provide some uncertainty) number of clinical cases of malaria averted per person per year that are predicted to be achievable by introducing an IRS campaign with different products, in addition to the existing bednet distribution. In all simulations it is assumed that 80% of the population is covered by the IRS campaign and that it is sprayed at the optimum moment prior to the local seasonal peak of transmission. Predictions are made for all administrative 1 units in Africa according to the level of seasonality (as measured by average rainfall) and historic LLIN coverage (as assessed in 2015[1]). Predictions are made for every site according to the level of pyrethroid resistance (as assessed by the percentage 24-hour survival of mosquitoes when exposed to the standard dose of pyrethroid in a discriminatory dose bioassay and monitored for 24-hours, ranging from 0%, all mosquitoes are killed, to 100%, all mosquitoes survive exposure). Parameter estimates that distinguish administration units can be provided on request. Numbers should be taken as approximate but dividing predicted costs of the programme (including procurement of product and coordination of spraying) by the number of cases averted provides a very simple estimate of the cost-effectiveness of the campaign for different products in that location.
